# Host-Pathogen Coevolution and the Emergence of Broadly Neutralizing Antibodies in Chronic Infections

**DOI:** 10.1371/journal.pgen.1006171

**Published:** 2016-07-21

**Authors:** Armita Nourmohammad, Jakub Otwinowski, Joshua B. Plotkin

**Affiliations:** 1 Joseph-Henri Laboratories of Physics and Lewis-Sigler Institute for Integrative Genomics, Princeton University, Princeton, New Jersey, United States of America; 2 Department of Biology, University of Pennsylvania, Philadelphia, Pennsylvania, United States of America; University of Chicago, UNITED STATES

## Abstract

The vertebrate adaptive immune system provides a flexible and diverse set of molecules to neutralize pathogens. Yet, viruses such as HIV can cause chronic infections by evolving as quickly as the adaptive immune system, forming an evolutionary arms race. Here we introduce a mathematical framework to study the coevolutionary dynamics between antibodies and antigens within a host. We focus on changes in the binding interactions between the antibody and antigen populations, which result from the underlying stochastic evolution of genotype frequencies driven by mutation, selection, and drift. We identify the critical viral and immune parameters that determine the distribution of antibody-antigen binding affinities. We also identify definitive signatures of coevolution that measure the reciprocal response between antibodies and viruses, and we introduce experimentally measurable quantities that quantify the extent of adaptation during continual coevolution of the two opposing populations. Using this analytical framework, we infer rates of viral and immune adaptation based on time-shifted neutralization assays in two HIV-infected patients. Finally, we analyze competition between clonal lineages of antibodies and characterize the fate of a given lineage in terms of the state of the antibody and viral populations. In particular, we derive the conditions that favor the emergence of broadly neutralizing antibodies, which may have relevance to vaccine design against HIV.

## Author Summary

We normally think of evolution occurring in a population of organisms, in response to their external environment. Rapid evolution of cellular populations also occurs within our bodies when the adaptive immune system works to eliminate infections. Some viruses, such as HIV, are able to evolve as quickly as our immune response, resulting in a chronic infection with both viral and immune populations perpetually adapting. Here we develop a mathematical description of this coevolutionary process, discover key parameters that govern the distribution of interactions between the two populations, introduce principled measures of adaptation, and analyze the conditions under which highly potent broadly neutralizing antibodies will emerge and dominate the immune response.

## Introduction

It takes decades for humans to reproduce, but our pathogens can reproduce in less than a day. How can we coexist with pathogens whose potential to evolve is 10^4^-times faster than our own? In vertebrates, the answer lies in their adaptive immune system, which uses recombination, mutation, and selection to evolve a response on the same time-scale at which pathogens themselves evolve.

**Fig 1:**
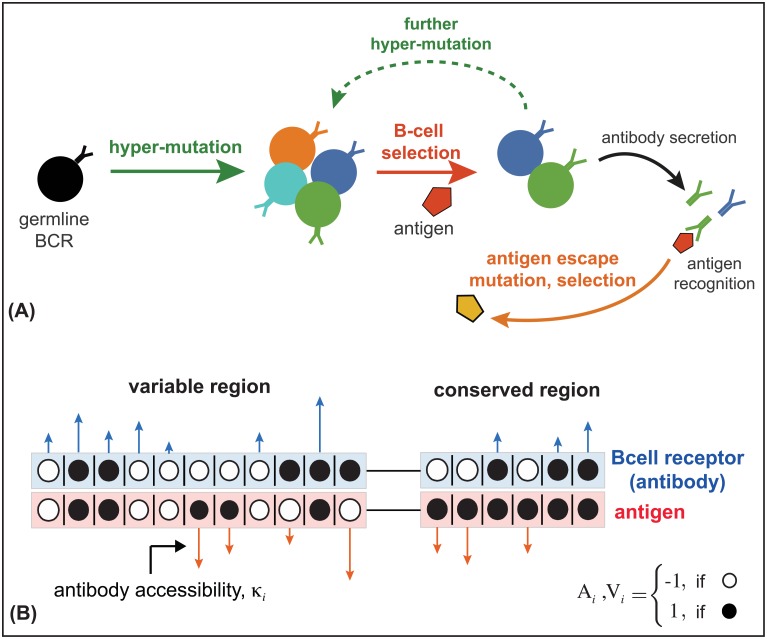
coevolution of antibodies and viruses. **(A)** Schematic of affinity maturation in a germinal center. A naive, germline B-cell receptor (black) with marginal binding affinity for the circulating antigen (red pentagon) enters the process of affinity maturation in a germinal center. Hypermutations produce a diverse set of B-cell receptors (colors), the majority of which do not increase the neutralization efficacy of B-cells, except for some beneficial mutations that increase binding affinity (dark blue and green) to the presented antigen. The selected B-cells may enter the blood and secrete antibodies, or enter further rounds of hypermutations to enhance their neutralization ability. Antigens mutate and are selected (yellow pentagon) based on their ability to escape the current immune challenge. **(B)** We model the interaction between the genotype of a B-cell receptor and its secreted antibody (blue) with a viral genotype (red) in both variable and conserved regions of the viral genome. The black and white circles indicate the state of the interacting loci with values ±1. Loci in the conserved region of the virus are fixed at +1. The length of the arrows indicate the contribution of each locus to the binding affinity, *κ*_*i*_, which is a measure of the accessibility of an antibody lineage to viral epitopes. The blue arrows indicate the interactions that increase binding affinity (i.e., loci with same signs in antibody and viral genotype), whereas red arrows indicate interaction that decrease the affinity (i.e., loci with opposite signs in antibody and viral genotype.)

One of the central actors in the adaptive immune system are B-cells, which recognize pathogens using highly diverse membrane-bound receptors. Naive B-cells are created by processes which generate extensive genetic diversity in their receptors via recombination, insertions and deletions, and hypermutations [1] which can potentially produce ∼10^18^ variants in a human repertoire [2]. This estimate of potential lymphocyte diversity outnumbers the total population size of B-cells in humans, i.e., ∼10^10^ [3, 4]. During an infection, B-cells aggregate to form *germinal centers*, where they hypermutate at a rate of about ∼10^−3^ per base pair per cell division over a region of 1-2 kilo base pairs [5]. The B-cell hypermutation rate is approximately 4–5 orders of magnitude larger than an average germline mutation rate per cell division in humans [6]. Mutated B-cells compete for survival and proliferation signals from helper T-cells, based on the B-cell receptor’s binding to antigens. This form of natural selection is known as *affinity maturation*, and it can increase binding affinities up to 10–100 fold [7–9], see Fig 1A. B-cells with high binding affinity may leave germinal centers to become antibody secreting plasma cells, or dormant memory cells that can be reactivated quickly upon future infections [1]. Secreted antibodies, which are the soluble form of B-cell receptors, can bind directly to pathogens to mark them for neutralization by other parts of the immune system. Plasma B-cells may recirculate to other germinal centers and undergo further hypermutation [8].

Some viruses, such as seasonal influenza viruses, evolve quickly at the population level, but the adaptive immune system can nonetheless remove them from any given host within a week or two. By contrast, chronic infections can last for decades within an individual, either by pathogen dormancy or by pathogens avoiding neutralization by evolving as rapidly as B-cell populations. HIV mutation rates, for example, can be as high as 0.1–0.2 per generation per genome [10]. Neutralizing assays and phylogenetic analyses suggest an evolutionary arms race between B-cells and HIV populations during infection in a single patient [11–15]. Viruses such as HIV have evolved to keep the sensitive regions of their structure inaccessible by the immune system e.g., through glycan restriction or immuno-dominant variable loops [16, 17]. As a result, the majority of selected antibodies bind to the most easily accessible regions of the virus, where viruses can tolerate mutations and thereby escape immune challenge. Nonetheless, a remarkably large proportion of HIV patients (∼20%) eventually produce antibodies that neutralize a broad panel of virions [18, 19] by attacking structurally conserved regions, such as the CD4 binding site of HIV *env* protein [14, 20–23]. These broadly neutralizing antibodies (BnAbs), can even neutralize HIV viruses from other clades, suggesting it may be possible to design an effective HIV vaccine if we can understand the conditions under which BnAbs arise [14, 20, 23–27].

Recent studies have focused on mechanistic modeling of germinal centers in response to one or several antigens [7, 28], and elicitation of BnAbs [27, 29]. However, these studies did not model the coevolution of the virus and B-cell repertoire, which is important to understand how BnAbs arise *in vivo*. Modeling of such coevolution is difficult because the mechanistic details of germinal center activity are largely unknown [15, 30], and the multitude of parameters make it difficult to identify generalizable aspects of a model. While evidence of viral escape mutations and B-cell adaptation has been observed experimentally [11–14] and modeled mechanistically [27, 29], it is not clear what are the generic features and relevant parameters in an evolutionary arms race that permit the development, or, especially, the early development of BnAbs. Phenomenological models ignore many details of affinity maturation and heterogeneity in the structure of germinal centers and yet produce useful qualitative predictions [15, 30, 31]. Past models typically described only a few viral types [27, 28], and did not account for the vast genetic diversity and turnover seen in infecting populations. A recent study by Luo & Perelson [30] described diverse viral and antibody populations, relying primarily on numerical simulations.

In this paper, we take a phenomenological approach to model the within-host coevolution of *diverse* populations of B-cells and chronic viruses. We focus on the chronic infection phase, where the immune response is dominated by HIV-specific antibody-mediated mechanisms, which follow the strong response by the cytotoxic T-lymphocytes (i.e., CD8+ killers T-cells), around 50 days after infection [32]. During the chronic phase, population sizes of viruses and lymphocytes are relatively constant but their genetic compositions undergo rapid turnover [33]. We characterize the interacting sites of B-cell receptors and viruses as mutable binary strings, with binding affinity, and therefore selection, defined by matching bits. We keep track of both variable regions in the viral genome and conserved regions, asking specifically when B-cell receptors will evolve to bind to the conserved region, i.e., to develop broad neutralization capacity. The main simplification that makes our analysis tractable is that we focus on the evolution of a shared interaction phenotype, namely the distribution of binding affinities between viral and receptor populations. Specifically, we model the effects of mutations, selection and reproductive stochasticity on the distribution of binding affinities between the two populations, which is similar to the approach of quantitative genetics [34]. Projecting from the high-dimensional space of genotypes to lower dimension of binding phenotypes allows for a predictive and analytical description of the coevolutionary process [35], whilst retaining the salient information about the quantities of greatest biological and therapeutic interest.

Using this modeling approach we show that the evolution of the binding affinity does not depend on details of any single-locus contribution, but is an emerging property of all constitutive loci. Even though the coevolution of antibodies and viruses is perpetually out of equilibrium, we develop a framework to quantify the amount of adaptation in each of the two populations by defining fitness and transfer flux, which partition changes in mean fitness. We discuss how to measure the fitness and transfer flux from time-shifted experiments, where viruses are competed against past and future antibodies, and we show how such measurements provide a signature of coevolution. We use these analytical results to interpret empirical measurements of time-shifted neutralization assays from two HIV-infected patients [11], and we infer two qualitatively different regimes of viral-antibody coevolution. We discuss the consequences of competition between clonal B-cell lineages within and between germinal centers. In particular, we derive analytic expressions for the fixation probability of a newly arisen, broadly neutralizing antibody lineage. We find that BnAbs have an elevated chance of fixation in the presence of a diverse viral population, whereas specific neutralizing antibody lineages do not. We discuss the implications of these results for the design of preventive vaccines that elicit BnAbs against HIV.

## Results

### Interaction between antibodies and viruses

B-cell receptors undergo mutation and selection in germinal centers, whereas viruses are primarily affected by the receptors secreted into the blood, known as antibodies. Our model does not distinguish between antibodies and B-cells, so we will use the terms interchangeably. To represent genetically diverse populations we define genotypes for antibodies and viruses as binary sequences of ±1, where mutations change the sign of individual loci. Mutations in some regions of a viral genome are highly deleterious, e.g. at sites that allow the virus to bind target cell receptors, including CD4-binding sites for HIV. To capture this property we explicitly model a conserved region of the viral genome that does not tolerate mutations, so that its bits are always set to +1. We let viruses have variable bits at positions *i* = 1 … ℓ, and conserved bits at positions $i=\ell +1,\ldots ,\ell +\hat{\ell }$; while antibodies have variable bits at positions $i=1\;\ldots \;\ell +\hat{\ell }$; see Fig 1B.

Naive B-cells generate diversity by gene rearrangements (VDJ recombination), which differentiates their ability to bind to different epitopes of the virus; and then B-cells diversify further by somatic hypermutation and selection during affinity maturation. We call the set of B-cells that originate from a common germline sequence a clonal lineage. A lineage with access to conserved regions of the virus can effectively neutralize more viral genotypes, since no escape mutation can counteract this kind of neutralization.

The binding affinity between antibody and virus determines the likelihood of a given antigen neutralization by an antibody, and therefore it is the key molecular phenotype that determines selection on both immune and viral populations. We model the binding affinity as a weighted dot product over all loci, which for antibody *A*^*α*^ chosen from the genotype space $\alpha \in 1\ldots 2^{\ell +\hat{\ell }}$ and virus *V*^*γ*^ with *γ* ∈ 1 … 2^*ℓ*^ has binding affinity

$$\begin{array}{ll} E_{tot}^{C}(A^{\alpha },V^{\gamma }) & =\underset{\text{variable viral region}}{\underset{︸}{\overset{\ell }{\underset{i=1}{\sum }}\kappa_{i}^{C}A_{i}^{\alpha }V_{i}^{\gamma }}}+\underset{\text{conserved viral region}}{\underset{︸}{\overset{\ell +\hat{\ell }}{\underset{i=\ell +1}{\sum }}{\hat{\kappa }}_{i}^{C}A_{i}^{\alpha }}}\\ & \equiv E_{\alpha \;\gamma }^{C}+{\hat{E}}_{\alpha }^{C} \end{array}$$ (1)

where, $A_{i}^{\alpha }=\pm 1$ denotes the *i*^*th*^ locus of the *α* antibody genotype, and $V_{i}^{\gamma }$ the *i*^*th*^ locus of the *γ* viral genotype. Matching bits at interacting positions enhance binding affinity between an antibody and a virus; see Fig 1B. Similar models have been used to describe B-cell maturation in germinal centers [27], and T-cell selection based on the capability to bind external antigens and avoid self proteins [36, 37]. The conserved region of the virus with *V*_*i*_ = 1 is located at positions $i=\ell +1,\ldots ,\ell +\hat{\ell }$ for all viral sequences. Consequently, the total binding affinity is decomposed into the interaction with the variable region of the virus, $E_{\alpha \;\gamma }^{C}$ and with the conserved region of the virus, ${\hat{E}}_{\alpha }^{C}$. We call the lineage-specific binding constants $\left\{\kappa_{i}^{C}\geq 0\right\}$ and $\left\{{\hat{\kappa }}_{i}^{C}\geq 0\right\}$ the *accessibilities*, because they characterize the intrinsic sensitivity of an antibody lineage to individual sites in viral epitopes. We begin by analyzing the evolution of a single antibody lineage, and suppress the C notation for brevity. Coevolution with multiple antibody lineages is discussed in a later section.

Both antibody and viral populations are highly polymorphic, and therefore contain many unique genotypes. While the binding affinity between a virus *V*^*γ*^ and an antibody *A*^*α*^ is constant, given by eq (1), the frequencies of the antibody and viral genotypes, *x*^*α*^ and *y*^*γ*^, and all quantities derived from them, change over time as the two populations coevolve. To characterize the distribution of binding affinities we define the genotype-specific binding affinities in each population, which are marginalized quantities over the opposing population: *E*_*α* ⋅_ = ∑_*γ*_ *E*_*αγ*_ *y*^*γ*^ for the antibody *A*^*α*^, and *E*_.*γ*_ = ∑_*α*_ *E*_*αγ*_ *x*^*α*^ for the virus *V*^*γ*^. We will describe the time evolution of the joint distribution of *E*_*α* ⋅_, ${\hat{E}}_{\alpha }$, and *E*_⋅ *γ*_, by considering three of its moments: (i) the mean binding affinity, which is the same for both populations $E=\sum_{\alpha }E_{\alpha \;\cdot }x^{\alpha }=\sum_{\gamma }E_{\cdot \;\gamma }y^{\gamma }$, (ii) the diversity of binding affinity in the antibodies, $M_{A,2}=\sum_{\alpha }(E_{\alpha \;\cdot }-E{)}^{2}x^{\alpha }$ and (iii) the diversity of binding affinities in the viruses, $M_{V,2}=\sum_{\gamma }(E_{\cdot \;\gamma }-E{)}^{2}y^{\gamma }$. Analogous statistics of binding affinities can be defined for the conserved region of the virus, which we denote by $\hat{E}$ for the mean interaction, and ${\hat{M}}_{A,2}$ for the diversity across antibodies. The diversity of viral interactions in the conserved region must always equal zero, ${\hat{M}}_{V,2}=0$.

### Coevolution of an antibody lineage and viruses

We first characterize the affinity maturation process of a single clonal antibody lineage coevolving with a viral population, which includes hypermutation, selection, and stochasticity due to population size in germinal centers, i.e., genetic drift.

#### Genetic drift and evolutionary time-scales

Stochasticity in reproductive success, known as genetic drift, is an important factor that depends on population size, and therefore we model genetic drift by keeping populations at finite size *N*_*a*_ for antibodies, and *N*_*v*_ for viruses. Although the population of B-cells can reach large numbers within an individual host, significant bottlenecks occur in germinal centers, where there may be on the order of ∼10^3^−10^4^ B-cells [7]. For HIV, estimates for intra-patient viral divergence suggests an effective population size of about ∼10^2^−10^3^, which is much smaller than the number of infected cells within a patient ∼10^7^−10^9^ [38].

Fluctuations by genetic drift define an important time-scale in the evolution of a polymorphic population: the neutral coalescence time is the characteristic time that two randomly chosen neutral alleles in the population coalesce to their most recent common ancestor, and is equal to *N* generations. Neutral coalescence time is estimated by phylogenetic analysis, and is often interpreted as an effective population size, which may be different from the census population size. Coalescence time can be mapped onto real units of time (e.g., days) if sequences are collected with sufficient time resolution. Without loss of generality, we assume that generation times in antibodies and viruses are equal, but we distinguish between the neutral coalescence time of antibodies and viruses by using distinct values for their population sizes, i.e., *N*_*a*_ in antibodies and *N*_*v*_ in viruses.

#### Mutations

In the bi-allelic model outlined in Fig 1B, a mutation changes the sign of an antibody site, i.e., $A_{i}^{\alpha }\to -A_{i}^{\alpha }$, affecting binding affinity in proportion to the lineage’s intrinsic accessibility at that site, *κ*_*i*_. Therefore, a mutation in an antibody at position *i* changes *E*_*α*._ by $\delta_{i}E_{\alpha \;.}=-2\;\kappa_{i}A_{i}^{\alpha }\sum_{\gamma }V_{i}^{\gamma }y^{\gamma }$. Likewise, a mutation at position *j* of a virus $V_{j}^{\gamma }\to -V_{j}^{\gamma }$ affects binding affinity in proportion to *κ*_*j*_. We assume constant mutation rates in the variable regions of the viruses and antibodies: *μ*_*v*_ and *μ*_*a*_ per site per generation.

Empirical estimates of per-generation mutation rates for viruses *μ*_*v*_ or hypermutation rates of BCR sequences *μ*_*a*_ are extremely imprecise, and so we rescale mutation rates by neutral coalescence times. To do this, we consider measurements of standing neutral sequence diversity, estimated from genetic variation in, e.g., four-fold synonymous sites of protein sequences at each position. Neutral sequence diversity for the antibody variable region, which spans a couple of hundred base pairs, is about *θ*_*a*_ = *N*_*a*_ *μ*_*a*_ = 0.05 − 0.1 [2]. Nucleotide diversity of HIV increases over time within a patient, and ranges between *θ*_*v*_ = *N*_*v*_ *μ*_*v*_ = 10^−3^ − 10^−2^ in the *env* protein of HIV-1 patients, with a length of about a thousand base pairs [39]. Interestingly, the total diversity of the variable region in BCRs is comparable to the diversity of its main target, the *env* protein, in HIV. Both populations have on the order of 1–10 mutations per genotype per generation, which we use as a guideline for parameterizing simulations of our model.

#### Selection

Frequencies of genotypes change according to their relative growth rate, or fitness. The change in the frequency of antibody *A*^*α*^ with fitness $f_{A^{\alpha }}$ is $\Delta x^{\alpha }=(f_{A^{\alpha }}-F_{A})x^{\alpha }$ per generation, where we define (malthusian) fitness as proportional to the growth rate, and $F_{A}=\sum_{\alpha }f_{A^{\alpha }}x^{\alpha }$ denotes the mean fitness of the antibody population (see Section A of S1 Text). Likewise, the change in frequency of virus *V*^*γ*^ due to selection per generation is, $\Delta y^{\gamma }=(f_{V^{\gamma }}-F_{V})y^{\gamma }$, where *F*_*V*_ denotes the mean fitness in the viral population.

During affinity maturation in a germinal center, a B-cell’s growth rate depends on its ability to bind to the limited amounts of antigen, and to solicit survival signals from helper T-cells [8]. At the same time, viruses are neutralized by antibodies that have high binding affinity. The simplest functional form that approximates this process, and for which we can provide analytical insight, is linear with respect to the binding affinity,

$$f_{A^{\alpha }}=S_{a}\left(E_{\alpha \;\cdot }+{\hat{E}}_{\alpha }\right)$$ (2)

$$f_{V^{\gamma }}=-S_{v}\left(E_{\cdot \;\gamma }+{\hat{E}}_{\cdot }\right)$$ (3)

for antibody *A*^*α*^ and virus *V*^*γ*^. The selection coefficient *S*_*a*_ > 0 quantifies the strength of selection on the binding affinity of antibodies. The value of *S*_*a*_ may decrease in late stages of a long-term HIV infection, as the host’s T-cell count decays [31], but we do not model this behavior. The viral selection coefficient *S*_*v*_ > 0 represents immune pressure impeding the growth of the virus. The contribution of the conserved region to the fitness of the virus is independent of the viral genotype in eq (3), and it does not affect the relative growth rates of the viral strains.

The number of sites and the magnitude of their accessibilities affect the overall strength of selection on binding affinity. Therefore, it is useful to absorb the intrinsic effects of the phenotype magnitude into the selection strength, and use rescaled values that are comparable across lineages of antibodies, and across experiments. We therefore rescale quantities related to the binding affinity by the total scale of the phenotypes $E_{0}^{2}=\sum_{i}\kappa_{i}^{2}$ and ${\hat{E}}_{0}^{2}=\sum_{i}{\hat{\kappa }}_{i}^{2}$, such that *E*_*αγ*_ → *E*_*αγ*_/*E*_0_ and ${\hat{E}}_{\alpha \gamma }\to {\hat{E}}_{\alpha \gamma }/{\hat{E}}_{0}$, resulting in rescaled mean binding affinities *ε* and $\hat{\varepsilon }$, and diversities *m*_*A*,2_, ${\hat{m}}_{A,2}$ and *m*_*V*,2_ in variable and conserved regions of both populations. Accordingly, we define rescaled selection coefficients *s*_*a*_ = *N*_*a*_ *S*_*a*_ *E*_0_, ${\hat{s}}_{a}=N_{a}S_{a}{\hat{E}}_{0}$, *s*_*v*_ = *N*_*v*_ *S*_*v*_ *E*_0_ and ${\hat{s}}_{v}=N_{v}S_{v}{\hat{E}}_{0}$, which describe the total strength of selection on binding affinity; see Section B.1 of S1 Text for details.

Many aspects of affinity maturation are not well known, and so it is worth considering other forms of selection. In Section B.5 of S1 Text we describe fitness as a non-linear function of the binding affinity. In particular, we consider fitness that depends on the antibody activation probability, which is a sigmoid function of the *strongest* binding affinity among a finite number of interactions with antigens. The linear fitness function in eq (2) is a limiting case of this more general fitness model. While most of our analytical results are based on the assumption of linear fitness function, we also discuss how to quantify adaptation for arbitrary fitness models, and we numerically study the effect of nonlinearity on the rate of antibody adaptation during affinity maturation.

### Evolution of the mean binding affinity

We focus initially on understanding the (rescaled) mean binding affinity *ε*, $\hat{\varepsilon }$ between a clonal antibody lineage and the viral population, since this is a proxy for the overall neutralization ability that is commonly monitored during an infection. Combining genetic drift with mutation and selection, and assuming a continuous-time and continuous-frequency process, results in a stochastic dynamical equation for the evolution of rescaled mean binding affinity in the variable region,

$$\frac{d}{d\tau }\varepsilon =-2\left[\theta_{a}+\theta_{v}\left(N_{A}/N_{v}\right)\right]\varepsilon +s_{a}m_{A,2}-s_{v}m_{V,2}+\sqrt{m_{A,2}+\frac{N_{a}}{N_{v}}m_{V,2}}\;\;\chi_{\varepsilon }$$ (4)

and in the conserved region,

$$\frac{d}{d\tau }\hat{\varepsilon }=-2\theta_{a}\hat{\varepsilon }+s_{a}{\hat{m}}_{A,2}+\sqrt{{\hat{m}}_{A,2}}\chi_{\hat{\varepsilon }}$$ (5)

where *χ*_*ε*_ and $\chi_{\hat{\varepsilon }}$ are standard Gaussian noise terms, and time *τ* is measured in units of the antibody coalescence time *N*_*a*_. Our analysis neglects the correlation between the variable and the conserved regions of the virus, which is due to physical linkage of the segments. In Section B.4 of S1 Text we show that a difference in evolutionary time-scales between these regions reduces the magnitude of this correlation. As eqs (4 and 5) reflect, mutations drive the mean affinity towards the neutral value, zero, whereas selection pushes it towards positive or negative values. The efficacy of selection on binding affinity is proportional to the binding diversity *m*_*A*,2_, *m*_*V*,2_ in each of the populations. If a population harbors a large diversity of binding affinities then it has more potential for adaptation from the favorable tail of the distribution, which contains the most fit individuals in each generation [40, 41]. It follows that selection on viruses does not affect the evolution of their conserved region, where the viral diversity of binding is always zero, ${\hat{m}}_{V,2}=0$. In Section B.3 of S1 Text and S2 Fig we study the evolution of the higher central moments in detail.

The dynamics in eqs (4 and 5) simplify in the regime where selection on individual loci is weak (*NSκ* < 1), but the additive effects of selection on the total binding affinity are substantial (1 ≲ *s* ≪ *θ*^−1^). This evolutionary regime is, in particular, relevant for HIV escape from the humoral neutralizing antibody response [39], that follows the initial strong response to cytotoxic T-lymphocytes [42]. In this parameter regime, the binding diversities are fast variables compared to the mean affinity, and can be approximated by their stationary ensemble-averaged values (S3 Fig), which depend only weakly on the strength of selection even for substantial selection *s* ∼ 1: 〈*m*_*A*,2_〉 ≃ 4*θ*_*a*_ and 〈*m*_*V*,2_〉 ≃ 4*θ*_*v*_. Higher-order corrections (Section B.3 of S1 Text and S2 Fig) show that strong selection reduces binding diversity. The ensemble-averaged mean binding affinities relax exponentially towards their stationary values,

$$\langle \varepsilon \rangle ≃\frac{2(s_{a}\theta_{a}-s_{v}\theta_{v}\left(N_{a}/N_{v}\right))}{\theta_{a}+\theta_{v}\left(N_{a}/N_{v}\right)}\equiv 2\;\Delta s_{av}$$ (6)

$$\langle \hat{\varepsilon }\rangle ≃2\;{\hat{s}}_{a}$$ (7)

where Δ*s*_*av*_ is an effective selection coefficient for binding affinity in the variable region, combining the effect of selection from both populations and accounting for their distinct genetic diversities. The stationary mean binding affinity quantifies the balance of mutation and selection acting on both populations. A strong selection difference between two populations Δ*s*_*av*_ ≫ 1 results in selective sweeps for genotypes with extreme values of binding affinity in each population, and hence, reduces the binding diversity. We validated our analytical solution for stationary mean binding, with corrections due to selection on binding diversity (Section B.3 of S1 Text), by comparison with full, genotype-based Wright-Fisher simulations across a broad range of selection strengths (Materials and Methods, S1 and S2 Figs).

**Fig 2:**
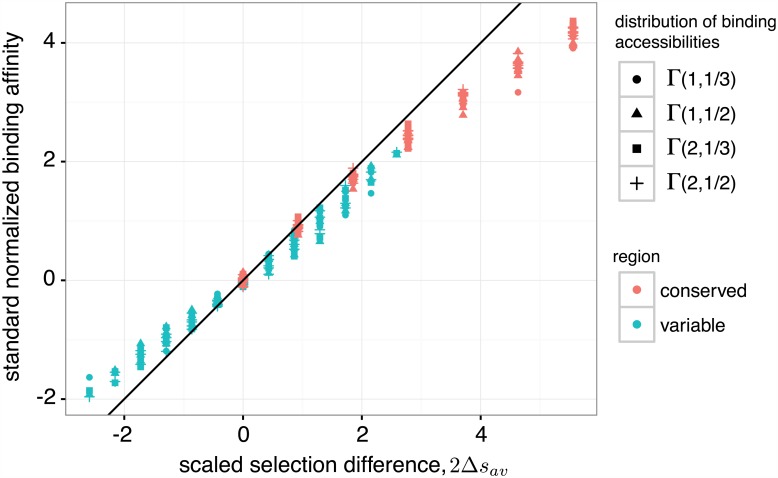
Effect of selection on immune-virus binding affinity. The stationary mean binding affinity, rescaled by antibody binding diversity ($E/\sqrt{M_{A,2}/4\theta_{a}}$), on the y-axis, is well approximated by the scaled selection difference between antibody and viral populations, Δ*s*_*av*_, as predicted by our analysis (eq (6)). Points show results of Wright-Fisher simulations, and the solid line has slope 1. Note that the mean binding affinity is insensitive to the details of heterogeneous binding accessibilities, *κ*_*i*_, associated with an antibody lineage. Accessibilities *κ*_*i*_ are drawn from several different Γ-distributions, shown in legend. Small deviations from the predicted mean binding are caused by higher moments of binding affinities, which can also be understood analytically (S1 Fig). Simulation parameters are detailed in the Materials and Methods.

The weak dependence of binding diversity on selection allows for an experimental estimation of the stationary rescaled mean binding affinity, using measurements of the binding affinity distribution and neutral sequence diversities. The rescaled binding affinity can be approximated as: $\varepsilon \approx \langle E\rangle /\sqrt{\langle M_{A,2}\rangle /4\theta_{a}}$ and $\hat{\varepsilon }\approx \langle \hat{E}\rangle /\sqrt{\langle {\hat{M}}_{A,2}\rangle /4\theta_{a}}$. Fig 2 demonstrates the utility of this approximation, and it shows that heterogeneous binding accessibilities, *κ*_*i*_, drawn from several different distributions, do not affect stationary mean binding. Only the total magnitude of the accessibilities is relevant, as it determines the effect of selection on the whole phenotype. Although we have formulated a high-dimensional stochastic model of antibody-antigen coevolution in polymorphic populations, we can nonetheless understand the long-term binding affinities, which are commonly measured in patients, in terms of only a few key parameters.

In Section B.5 of S1 Text we numerically study non-linear fitness landscapes, and their effect on the stationary mean binding and rate of adaptation (S4 Fig). While the results differ quantitatively, we can qualitatively understand how the stationary mean binding affinity depends on the form of non-linearity.

### Fitness and transfer flux

The antagonistic coevolution of antibodies and viruses is a non-equilibrium process, with each population constantly adapting to a dynamic environment, namely, the state of the opposing population. As a result, any time-independent quantity, such as the stationary mean binding affinity studied above, is itself not informative for the extent of coevolution that is occurring. For example, a stationary mean binding affinity of zero (equivalently Δ*s*_*av*_ = 0 in eq (6)) can indicate either neutral evolution or rapid coevolution induced by equally strong selection in antibody and viral populations.

**Fig 3:**
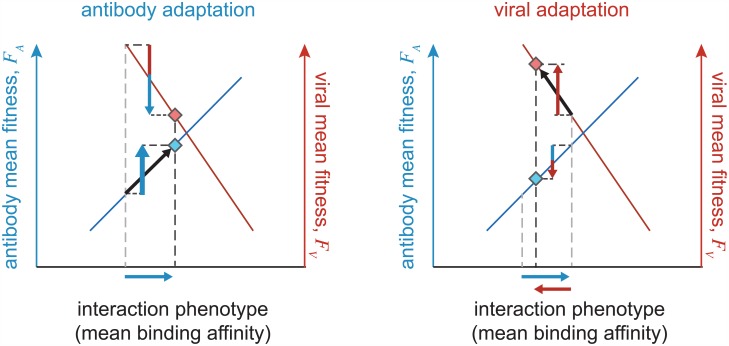
Fitness and transfer flux in antibody-viral coevolution. The schematic diagram shows adaptation of antibody (blue diamond) and viral (red diamond) populations on their respective fitness landscapes, which depend on the common binding phenotype shown on the x-axis (i.e., the mean binding affinity). During one step of antibody adaptation (left), mean binding affinity increases (horizontal blue arrow) to enhance the fitness of the antibody population, with a rate equal to the antibody fitness flux *ϕ*_*A*_ (upward blue arrow). In the regime of strong selection, the fitness flux is proportional to the variance of fitness in the population; see eq (8). Adaptation of antibodies reduces the mean fitness in the viral population, with a rate proportional to the transfer flux from antibodies to viruses $T_{A\to V}$ (downward red/blue arrow). On the other hand, viral adaptation (right) reduces the binding affinity and affects the fitness of both populations, with rates proportional to the viral fitness flux *ϕ*_*V*_ (upward red arrow) and the transfer flux from viruses to antibodies $T_{V\to A}$ (downward blue/red arrow); see eq (9). Cumulative fitness flux (the sum of upward arrows) and cumulate transfer flux (the sum of downward arrows) over an evolutionary period quantify the amount of adaptation and interaction in the two antagonistic populations.

To quantify the amount of adaptation and extent of interaction in two coevolving populations we will partition the change in mean fitness of each population into two components. We measure adaptation by the *fitness flux* [43–45], which generically quantifies adaptation of a population in response to a changing environment (in this case the opposing population); see schematic Fig 3. For our model, the fitness flux of the antibody population quantifies the effect of changing genotype frequencies on mean fitness, and is defined as $\phi_{A}(t)=\sum_{\alpha }\partial_{x^{\alpha }}F_{A}(t)dx^{\alpha }(t)/dt$, where *F*_*A*_ denotes the mean fitness of antibodies, and the derivative *dx*^*α*^(*t*)/*dt* measures the change in frequency of the antibody *A*^*α*^. The forces of mutation, drift, and selection all contribute to fitness flux, however the portion of fitness flux due to selection equals the population variance of fitness, in accordance with Fisher’s theorem [40]. The second quantity we study, which we term the *transfer flux*, measures the amount of interaction between the two populations by quantifying the change in mean fitness due to the response of the opposing population (schematic Fig 3). The transfer flux from viruses to antibodies is defined as $T_{V\to A}(t)=\sum_{\gamma }\partial_{y^{\gamma }}F_{A}(t)dy^{\gamma }(t)/dt$. Analogous measures of adaptation and interaction can be defined for the viral population (see Section C of S1 Text).

The fitness flux and transfer flux represent rates of adaptation and interaction, and they are typically time dependent, except in the stationary state. The total amount of adaptation and interaction during non-stationary evolution, where the fluxes change over time, can be measured by the cumulative fluxes over a period of time: $\Phi_{A}\left(\tau_{a}\right)=N_{a}\int_{t^{\prime }=0}^{t}\phi_{A}\left(t^{\prime }\right)\;dt^{\prime }$ and $T_{V\to A}\left(\tau_{a}\right)=N_{a}\int_{t^{\prime }=0}^{t}T_{V\to A}\left(t^{\prime }\right)\;dt^{\prime }$, where time *τ*_*a*_ = *t*/*N*_*a*_ is measured in units of neutral coalescence time of antibodies *N*_*a*_. In the stationary state, the ensemble-averaged cumulative fluxes grow linearly with time. For coevolution on the fitness landscapes given by eqs (2 and 3), the ensemble-averaged, stationary cumulative fitness flux and transfer flux in antibodies are

$$\langle \Phi_{A}\left(\tau_{a}\right)\rangle =\left[-2\theta_{a}s_{a}\langle \varepsilon \rangle +s_{a}^{2}\langle m_{A,2}\rangle \right]\tau_{a}$$ (8)

$$\langle T_{V\to A}\left(\tau_{a}\right)\rangle =\left[-2\theta_{v}s_{a}\langle \varepsilon \rangle -s_{a}s_{v}\langle m_{V,2}\rangle \right]\left(N_{a}/N_{v}\right)\tau_{a}$$ (9)

Note that the factor (*N*_*a*_/*N*_*v*_)*τ*_*a*_ in eq (9), which is a rescaling of time in units of viral neutral coalescence time *τ*_*v*_ = *t*/*N*_*v*_, emphasizes the distinction between the evolutionary time scales of antibodies and viruses. The first terms on the right hand side of eqs (8 and 9) represent the fitness changes due to mutation, the second terms are due to selection, and the effects of genetic drift are zero in the ensemble average for our linear fitness landscape. Notably, the flux due to the conserved region of the virus is zero in stationarity, as is the case for evolution in a static fitness landscape (i.e., under equilibrium conditions). In the stationary state, the cumulative fitness and transfer fluxes sum up to zero, 〈Φ_*A*_(*τ*_*a*_)〉 + 〈**T**_*V*→*A*_(*τ*_*a*_)〉 = 0.

Fitness flux and transfer flux are generic quantities that are independent of the details of our model, and so they provide a natural way to compare the rate of adaptation in different evolutionary models or in different experiments. In the regime of strong selection *s*_*a*_, *s*_*v*_ ≳ 1, non-linearity of the fitness function results in a more narrow distribution of fitness values in the antibody population, and hence, reduces the rate of adaptation and fitness flux; see S4 Fig. In the following section we show how to use fitness and transfer flux to detect signatures of significant antibody-antigen coevolution.

### Signature of coevolution and inferences from time-shifted experiments

Measuring interactions between antibodies and viruses isolated from different times provides a powerful way to identify coevolution. These “time-shifted” neutralization measurements in HIV patients have shown that viruses are more resistant to past antibodies, from which they have been selected to escape, and more susceptible to antibodies from the future, due to selection and affinity maturation of B-cells [11–13].

**Fig 4:**
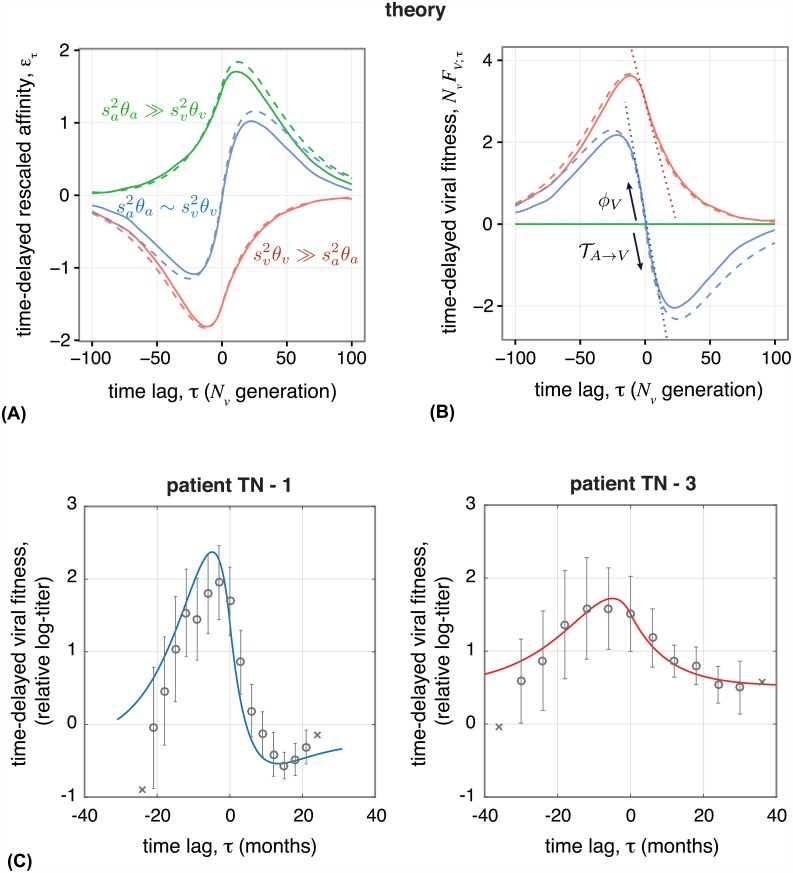
Time-shifted binding assays between antigens and antibodies provide a definitive signature of viral-immune coevolution. Viruses perform best against antibodies from the past and perform worst against antibodies from the future due to the adaptation of antibodies. **(A)** Stationary rescaled binding affinity between viruses from time *t* and antibodies from time *t* + *τ*, averaged over all t: *ε*_*τ*_ = 〈∑_*α*,*γ*_ *E*_*αγ*_ *y*^*γ*^(*t*)*x*^*α*^(*t* + *τ*)〉_*t*_/*E*_0_, and **(B)** time-shifted mean fitness of viruses *N*_*v*_ *F*_*V*;*τ*_ = −*s*_*v*_ *ε*_*τ*_, are shown for three regimes of coevolutionary dynamics: strong adaptation of both populations $s_{a}^{2}\theta_{a}∼s_{v}^{2}\theta_{v}$, with *s*_*a*_ = *s*_*v*_ = 2 (blue), stronger adaptation of viruses $s_{v}^{2}\theta_{v}\gg s_{a}^{2}\theta_{a}$ with *s*_*v*_ = 2, *s*_*a*_ = 0 (red), and stronger adaptation of antibodies $s_{a}^{2}\theta_{a}\gg s_{v}^{2}\theta_{v}$ with *s*_*v*_ = 0, *s*_*a*_ = 2 (green). Wright-Fisher simulations (solid lines) are compared to the analytical predictions from eqs. (S102, S103) in S1 Text for each regime (dashed lines). The “S”-shape curve in the blue regime is a signature of two antagonistically coevolving populations *s*_*v*_ *θ*_*v*_ ∼ *s*_*a*_ *θ*_*a*_. For large time-shifts, binding relaxes to its neutral value, zero, as mutations randomize genotypes. In the absence of selection in one population, the time-shifted binding affinity reflects adaptation of one population against stochastic variation in the other due to mutation and genetic drift. The slope of time-shifted fitness at lag *τ* = 0 is the viral population’s fitness flux (slope towards the past) and the transfer flux from the opposing population (slope towards the future), which are equal to each other in the stationary state. The slope of the dotted lines indicate the predicted fitness flux and transfer flux (eqs. (8 and 9)). Time-shifted fitness shown here does not include binding to the conserved region since that value is constant for all time-shifts in stationarity (see S6 Fig for non-stationary state). Simulation parameters are given in the Materials and Methods. **(C)** Empirical time-shifted fitness measurements of HIV based on a neutralization titer (IC_50_) [11], averaged over all time points with equal time-shift *τ*. Circles show averaged fitness ± 1 standard error, and crosses show fitness at time-shifts with only a single data point. Solid lines show analytical fits of our model to the data (see Materials and Methods and Section F of S1 Text). In patient TN-1, viruses and antibodies experience a comparable adaptive pressure, with a similar time-shift pattern to the blue “S-curve” in panel (B). In patient TN-3, however, adaptation in viruses is much stronger than in antibodies, resulting in an imbalanced shape of the time-shifted fitness curve, similar to the red curve in panel (B).

We can predict the form of time-shifted binding assays under our model; see Section D of S1 Text for details. The rescaled time-shifted binding affinity between viruses at time *t* and antibodies at time *t* + *τ* is given by *ε*_*τ*_(*t*) = ∑_*α*,*γ*_ *E*_*αγ*_ *y*^*γ*^(*t*)*x*^*α*^(*t* + *τ*)/*E*_0_ and ${\hat{\varepsilon }}_{\tau }\left(t\right)=\sum_{\alpha }{\hat{E}}_{\alpha }x^{\alpha }\left(t+\tau \right)/{\hat{E}}_{0}$ for the variable and the conserved region, respectively. The corresponding viral mean fitness at time *t* against the antibody population at time *t* + *τ* is $N_{v}F_{V;\tau }\left(t\right)=-s_{v}\left(\varepsilon_{\tau }\left(t\right)+{\hat{\varepsilon }}_{\tau }\left(t\right)\right)$. The slope of the time-shifted viral fitness at the time where the two populations co-occur (i.e., *τ* = 0), approaching from negative *τ*, i.e., from the past, measures the amount of adaptation of the viral population in response to the state of the antibody population, and it is precisely equal to the fitness flux of viruses: ∂_*τ*_ *F*_*V*;*τ*_(*t* − *τ*)|_*τ* = 0^−^_ = *ϕ*_*V*_(*t*). The slope approaching from positive time-shifts, i.e., from the future, measures the change in the mean fitness of the viral population due to adaptation of the antibody population, and it is precisely equal to the transfer flux from antibodies to viruses $\partial_{\tau }F_{V;\tau }(t){|}_{\tau =0^{+}}=\;T_{A\to V}(t)$. Similarly, we can define time-shifted fitness with antibodies as the focal population; see Section D of S1 Text. In stationarity, the sum of fitness flux and transfer flux is zero on average, and so the slopes from either side of *τ* = 0 are equal, as in Fig 4 and S5 Fig. Note that these relationships between time-shifted fitness and the flux variables hold in general, beyond the specific case of a linear fitness landscape. In a non-stationary state, the fitness flux and transfer flux are not balanced, and so 〈*F*_*V*;*τ*_(*t*)〉 has a discontinuous derivative at *τ* = 0 (S6 Fig). Therefore, observation of such a discontinuity provides a way to identify stationarity versus transient dynamics, given sufficient replicated experiments.

Whether in stationarity or not, the signature of out-of-equilibrium evolution is a positive fitness flux and negative transfer flux. For time-shifted fitness, this means that for short time shifts, where dynamics are dominated by selection, viruses have a higher fitness against antibodies from the past, and have lower fitness against antibodies from the future. This is true even when one population is evolving neutrally and the other has substantial selection, as shown in Fig 4A. For long time shifts, the sequences are randomized by mutations and the fitness decays exponentially to the neutral value. When selection and mutation are substantial on both sides the time-shifted fitness curve has a characteristic “S”shape—a signature of coevolution, whose inflected form can be understood in terms of the fitness and transfer fluxes. In Section D of S1 Text we analytically derive the functional form of the time-shifted binding affinity and fitness dependent on the evolutionary parameters. Fig 4A and 4B and S5 Fig show good agreement between Wright-Fisher simulations and our analytical predictions given by eqs. (S102, S103) in S1 Text for the stationary time-shifted binding affinity and viral fitness.

We can use our analytical results to interpret empirical measurements of time-shifted viral neutralization by a patient’s circulating antibodies. We analyzed data from Richman *et al.* [11] on two HIV-infected patients. We approximated the fitness of the virus against a sampled serum (antibodies) as the logarithm of the neutralization titer *F*_*V*_ ≃ −log titer; here titer is the reciprocal of antibody dilution where inhibition reaches 50% (IC_50_) [46]. A signature of coevolution can sometimes be obscured when the fitnesses of antibodies and viruses also depend on time-dependent intrinsic and environmental factors, such as drug treatments [46]. Therefore, we used fitness of a neutralization-sensitive virus (NL43) as a control measurement to account for the increasing antibody response during infection, shown in S7 Fig. The relative time-shifted viral fitness in Fig 4C for the two HIV patients (TN-1 and TN-3), match well with the fits of our analytical equations (see Materials and Methods and Section F of S1 Text). The inferred parameter values indicate two distinct regimes of coevolutionary dynamics in the two patients. In patient TN-1, viruses and antibodies experience a comparable adaptive pressure, as indicated by the “S-curve” in Fig 4C (blue line), whereas in patient TN-3, adaptation in viruses is much stronger than in antibodies, resulting in an imbalanced shape of the time-shifted fitness curve in Fig 4C (red line). We describe the inference procedure and report all inferred parameters in Section F of S1 Text. The resolution of the data [11] allows only for a qualitative interpretation of coevolutionary regimes. A more quantitative analysis can be achieved through longer monitoring of a patient, detailed information on the inhibition of viral replication at various levels of antibody dilution, and directed neutralization assays against HIV-specific antibody lineages.

### Competition between multiple antibody lineages

B-cells in the adaptive immune system are associated with clonal lineages that originate from distinct ancestral naive cells, generated by germline rearrangements (VDJ recombination) and junctional diversification [1]. Multiple lineages may be stimulated within a germinal center, and also circulate to other germinal centers [8]. Lineages compete for activation agents (e.g., helper T-cells) and interaction with a finite number of presented antigens [8]. We extend our theoretical framework to study how multiple lineages compete with each other and coevolve with viruses. This generalization allows us to show that lineages with higher overall binding ability, higher fitness flux, and lower (absolute) transfer flux have a better chance of surviving. In particular, we show that an antibody repertoire fighting against a highly diversified viral population, e.g., during late stages of HIV infection, favors elicitation of broadly neutralizing antibodies compared to normal antibodies.

**Fig 5:**
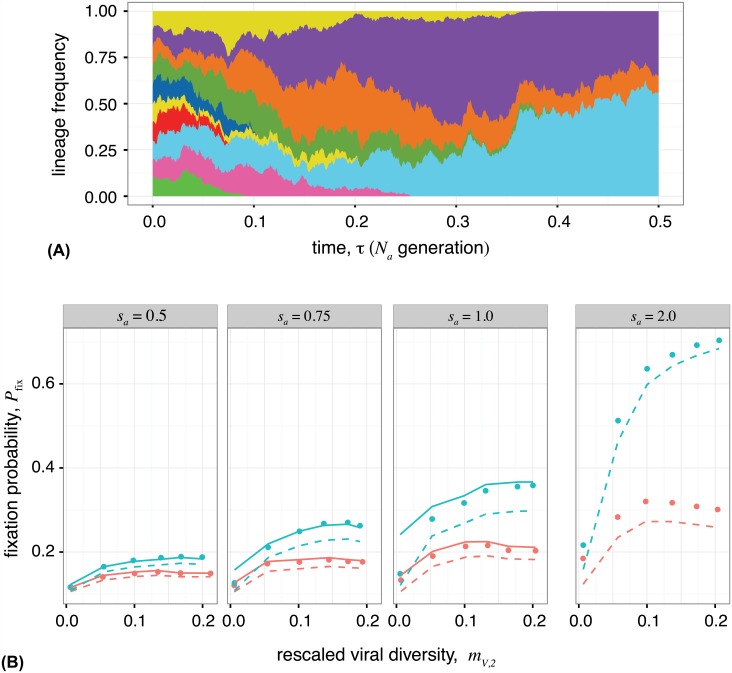
Competition between antibody lineages, and fixation of broadly neutralizing antibodies. **(A)** Simulation of competition between 20 clonal antibody lineages against a viral population. Lineages with higher mean fitness, higher fitness flux, and lower transfer flux tend to dominate the antibody repertoire. Each color represents a distinct antibody lineage, however there is also diversity within each lineage from somatic hypermutations. The reduction in the number of circulating lineages resembles the reduction in the number of active B-cell clones within the life-time of a germinal center [8]. Lineages are initialized as 500 random sequences with random accessibilities $\kappa^{C}$’s, unique to each lineage, drawn from an exponential distribution with rate parameter 3. Total population sizes are *N*_*a*_ = *N*_*v*_ = 10^4^. Other simulation parameters are as specified in the Materials and Methods. **(B)** Analytical estimates of the fixation probability *P*_fix_ of a new antibody lineage, based on the state of the populations at the time of its introduction, compared to Wright-Fisher simulations (points) with two competing antibody lineages. A novel BnAb (blue) or non-BnAb (red) lineage is introduced at frequency 10% into a non-BnAb resident population (simulation procedures described in the Materials and Methods). BnAb lineages have a higher chance of fixing, compared to non-BnAb antibodies, when the viral population is diverse, whereas both types of Abs have similar chances in the limit of low viral diversity. The solid line is the analytical estimate for *P*_fix_ given by eq. (S140) in S1 Text, which is valid when the rate of adaptation is similar in antibodies and viruses. The dashed line is the analytical estimate for *P*_fix_ using the approximation in eq. (S141) in S1 Text, which is suitable when there is a strong imbalance between the two populations, as is the case for invasion of a BnAb with strong antibody selection *s*_*a*_ > 1 or against a viral population with low diversity. In the absence of selection (neutral regime), the fixation probability of an invading lineage is equal to its initial frequency of 10%. Panels show different strengths of antibody selection *s*_*a*_ = 0.5, 0.75, 1,2 against a common viral selection strength *s*_*v*_ = 1. Viral diversity is influenced mostly by the viral nucleotide diversity *θ*_*v*_, which ranges from 0.002 to 0.1. Other simulation parameters are specified in the Materials and Methods.

The binding preference of a clonal antibody lineage C to the viral sequence is determined by its site-specific accessibilities $\left\{\kappa_{i}^{C},{\hat{\kappa }}_{i}^{C}\right\}$, defined in Fig 1B. The distribution of site-specific accessibilities over different antibody lineages $P_{C}(\{\kappa_{i}^{C},{\hat{\kappa }}_{i}^{C}\})$ characterizes the ability of an antibody repertoire to respond to a specific virus. Without continual introduction of new lineages, one lineage will ultimately dominate and the rest will go extinct within the coalescence time-scale of antibodies, *N*_*a*_ (Fig 5A). In reality, constant turn-over of lineages results in a highly diverse B-cell response, with multiple lineages acting simultaneously against an infection [47].

Stochastic effects are significant when the size of a lineage is small, so an important question is to find the probability that a low-frequency antibody lineage reaches an appreciable size and fixes in the population. We denote the frequency of an antibody lineage with size $N_{a}^{C}$ by $\rho^{C}=N_{a}^{C}/N_{a}$. The growth rate of a given lineage C depends on its relative fitness $F_{A^{C}}$ compared to the rest of the population,

$$\frac{d}{dt}\rho^{C}=\left(F_{A^{C}}-F_{A}\right)\rho^{C}+\sqrt{\frac{\rho^{C}\left(1-\rho^{C}\right)}{N_{a}}}\chi_{{}_{C}}$$ (10)

where $F_{{}_{A}}=\sum_{C}F_{A^{C}}\rho^{C}$ is the average fitness of the entire antibody population, and $\chi_{{}_{C}}$ is a standard Gaussian noise term. For the linear fitness landscape from eq (2), the mean fitness of lineage C is $F_{A^{C}}=S_{a}\left(E^{C}+{\hat{E}}^{C}\right)$. The probability of fixation of lineage C equals the asymptotic (i.e., long time) value of the ensemble-averaged lineage frequency, $P_{\text{fix}}\left(C\right)=\lim_{t\to \infty }\langle \rho^{C}\left(t\right)\rangle$.

Similar to evolution of a single lineage, the dynamics of a focal lineage are defined by an infinite hierarchy of moment equations for the fitness distribution. In the regime of substantial selection, and by neglecting terms due to mutation, a suitable truncation of the moment hierarchy allows us to estimate the long-time limit of the lineage frequency, and hence, its fixation probability (see Section E of S1 Text). For an arbitrary fitness function, fixation probability can be expressed in terms of the ensemble-averaged relative mean fitness, fitness flux and transfer flux at the time of introduction of the focal lineage,

$$\begin{array}{l} P_{\text{fix}}\left(C\right)/P_{0_{\text{fix}}}≃1+\langle N_{a}\left(F_{A^{C}}\left(0\right)-F_{A\left(0\right)}\right)\rangle \\ +\frac{N_{a}^{2}}{3}\langle \phi_{A^{C}}\left(0\right)-\phi_{A}\left(0\right)\rangle -N_{a}N_{v}\langle \left|T_{V\to A^{C}}\left(0\right)\left|-\right|T_{V\to A}\left(0\right)\right|]\rangle \end{array}$$ (11)

where *P*_0_fix__ is the fixation probability of the lineage in neutrality, which equals its initial frequency at the time of introduction, $P_{0_{\text{fix}}}=\rho^{C}\left(0\right)$. The first order term that determines the excess probability for fixation of a lineage is the difference between its mean fitness and the average fitness of the whole population. Thus, a lineage with higher relative mean fitness at the time of introduction, e.g., due to its better accessibility to either the variable or conserved region, will have a higher chance of fixation. Moreover, lineages with higher rate of adaptation, i.e., fitness flux $\phi_{A^{C}}\;(t=0)$, and lower (absolute) transfer flux from viruses $\left|T_{V\to A^{C}}\left(t=0\right)\right|$ tend to dominate the population.

For evolution in the linear fitness landscape, we can calculate a more explicit expansion of the fixation probability that includes mutation effects. In this case, the fixation probability of a focal lineage can be expressed in terms of the experimentally observable lineage-specific moments of the binding affinity distribution, instead of the moments of the fitness distribution (see Section E of S1 Text).

### Emergence of broadly neutralizing antibodies

With our multi-lineage model, we can understand the conditions for emergence of broadly neutralizing antibodies (BnAbs) in an antibody repertoire. Similar to any other lineage, the progenitor of a BnAb faces competition with other resident antibody lineages that may be dominating the population. The dominant term in the fixation probability is the relative fitness difference of the focal lineage to the total population at the time of introduction. Lineages may reach different fitnesses because they differ in their scale of interaction with the viruses, $E_{0}^{C}$ in the variable region and ${\hat{E}}_{0}^{C}$ in the conserved region; see Section E of S1 Text for details. Lineages which bind primarily to the conserved region, i.e., ${\hat{E}}_{0}^{C}\gg E_{0}^{C}$, are not vulnerable to viral escape mutations that reduce their binding affinity. Such BnAbs may be able to reach higher fitnesses compared to normal antibodies which bind to the variable region with a comparable scale of interaction. The difference in the mean fitness of the two lineages becomes even stronger, when viruses are more diverse (i.e., high *M*_*V*,2_), so that they can strongly compromise the affinity of the lineage that binds to the variable region; see eq (11).

If the invading lineage has the same fitness as the resident lineage, then the second order terms in eq (11) proportional to the fitness and transfer flux may be relevant. A BnAb lineage that binds to the conserved region has a reduced transfer flux than a normal antibody lineage, all else being equal. The difference in transfer flux of the two lineages depends on the viral diversity *M*_*V*,2_, and becomes more favorable for BnAbs when the viral diversity is high. Overall, a BnAb generating lineage has a higher advantage for fixation compared to normal antibodies, when the repertoire is coevolving against a highly diversified viral population, e.g., during late stages of HIV infection.

In Fig 5B we compare the fixation probability of a BnAb lineage, that binds only to the conserved region, with a normal antibody lineage that binds only to the variable region. In both cases we assume that the emerging lineage competes against a resident population of normal antibodies. We compare our analytical predictions for fixation probability as a function of the initial state of the antibody and viral populations given by eqs. (S140, S141) in S1 Text, with Wright-Fisher simulations of coevolving populations (numerical procedures detailed in the Materials and Methods). Increasing viral diversity *M*_2,*V*_ increases the fixation of BnAbs, but does not influence fixation of normal lineages. For low viral diversity, fixation of BnAbs is similar to normal Abs, and therefore they might arise and be outcompeted by other antibody lineages.

## Discussion

We have presented an analytical framework to describe coevolutionary dynamics between two antagonistic populations based on molecular interactions between them. We have focused our analysis on antibody-secreting B-cells and chronic infections, such as HIV. We identified effective parameters for selection on B-cells during hypermutation that enhance their binding and neutralization efficacy, and conversely parameters for selection on viruses to escape antibody binding. The resulting “red-queen” dynamics between antibodies and viruses produces a characteristic signature of coevolution in our model, i.e., viruses are resistant to antibodies from the past and are susceptible to antibodies from the future. We used our results to infer modes of immune-viral coevolution based on time-shifted neutralization measurements in two HIV-infected patients. Finally, we have shown that emergence and fixation of a given clonal antibody lineage is determined by competition between circulating antibody lineages, and that broadly neutralizing antibody lineages, in particular, are more likely to dominate in the context of a diverse viral population.

Luo and Perelson [30] found that competition between lineages caused BnAbs to appear late in their simulations. In addition, they found that multiple viral founder strains dilutes the competition of BnAbs with specific antibodies, leading to higher chance of BnAb appearance. The assumptions of their simulations differ in many ways from those of our model, and yet their overall finding agrees with our analytical results: BnAbs fix more readily when there is a large diversity of viral binding. In contrast to Luo and Perelson’s simulations which made assumptions about the immunogenicity of BnAbs, our analytic results show explicitly how differences in fitness of antibodies and the efficacy of viral escape affect competition between antibody lineages.

Our model is simple enough to clarify some fundamental concepts of antibody-antigen dynamics. However, understanding more refined aspects of B-cell-virus coevolution will require adding details specific to affinity maturation and viral reproduction, such as non-neutralizing binding between antibodies and antigens [15, 48], epitope masking by antibodies [49] and spatial structure of germinal centers [8]. Importantly, viral recombination [38, 39, 50] and latent viral reservoirs [51] are also known to influence the evolution of HIV within a patient. Similarly, the repertoire of the memory B-cells and T-cells, which effectively keep a record of prior viral interactions, influence the response of the adaptive immune system against viruses with antigenic similarity.

While our analysis has focused on coevolution of chronic viruses with the immune system, our framework is general enough to apply to other systems, such as bacteria-phage coevolution. Likewise, the notions of fitness and transfer flux as measures of adaptation are independent of the underlying model. Bacteria-phage interactions have been studied by evolution experiments [52, 53], and by time-shifted assays of fitness [54, 55], but established models of coevolution typically describe only a small number of alleles with large selection effects [56]. In contrast, our model offers a formalism for bacteria-phage coevolution where new genotypes are constantly produced by mutation, consistent with experimental observations [54]. Similarly, our formalism may be applied to study the evolution of seasonal influenza virus in response to the “global” immune challenge, imposed by a collective immune landscape of all recently infected or vaccinated individuals. Time-shifted binding assays of antibodies to influenza surface proteins are already used to gauge the virulence and cross-reactivity of viruses [57]. Quantifying the fitness flux and transfer flux, based on these assays, is therefore a principled way to measure rates of immunologically important adaptation in these systems.

One central challenge in HIV vaccine research is to devise a means to stimulate a lineage producing broadly neutralizing antibodies. Common characteristics of BnAbs, such as high levels of somatic mutation or large insertions, often lead to their depletion by mechanisms of immune tolerance control [58]. Therefore, one strategy to elicit these antibodies is to stimulate the progenitors of their clonal lineage, which may be inferred by phylogenetic methods [59], and to guide their affinity maturation process towards a broadly neutralizing state. Understanding the underlying evolutionary process is necessary to make principled progress towards such strategies, and this study represents a step in that direction. For example, our results suggest that a vaccine based on a genetically diverse set of viral antigens is more likely to stimulate BnAbs.

## Materials and Methods

### Simulations

Simulations of the full genotype model (Wright-Fisher dynamics) were implemented as follows. Viral and antibody populations consist of genotypes as strings of ±1 with length $\ell +\hat{\ell }$. Binding interactions are calculated between all pairs of antibodies and viruses as in eq (1), which define the fitness as in eqs (2 and 3). Genotypes within an antibody lineage share the same accessibilities, $\left\{\kappa_{i},\;{\hat{\kappa }}_{i}\right\}$. For each generation, a poisson distributed number of mutations occur, with each mutation flipping the sign of a site. Each generation is replaced by their offspring which inherit their parents’ genotype. Each parent generates a binomially distributed number of offspring, with probability proportional to the exponential of its fitness, with the constraint that the total number of individuals remains constant *N*_*a*_ in antibodies and *N*_*v*_ in viruses, which is equivalent to multinomial sampling. Note that we define fitness as “malthusian”, which means that fitness is the relative growth rate of genotypes, and the expected number of offspring is proportional to the exponential of fitness.

Simulation parameters for all figures are *N*_*a*_ = *N*_*v*_ = 10^3^, $\ell =\hat{\ell }=50$, *θ*_*a*_ = *θ*_*v*_ = 1/50, and all $\kappa_{i}={\hat{\kappa }}_{i}=1$, unless otherwise stated. Populations are initialized with all individuals having the same randomly generated genotype. To measure quantities in the stationary state (Figs 2 and 4) simulations are run for 10^4^ *N*_*a*_ generations, and quantities are averaged from samples every *N*_*a*_ generations. Data from the beginning of the simulations are omitted from the calculations, where the cutoff is $\tau =2\mu_{a}^{-1}$, the correlation time for the mean binding (S3 Fig and Section B.4 of S1 Text). To produce the simulations shown in Fig 5B, the newly emerging antibody lineages compete with the resident population as follows. First, the resident lineage is evolved with the virus for 50*N*_*a*_ generations to build up diversity. Simultaneously, the invading lineage is evolved with the virus, except that the viral fitness is determined only by the resident lineage. This ensures that invading lineages can marginally bind to the viral population, and are functional lineage progenitors; a process that happens prior to affinity maturation in germinal centers. The pre-adaptation of the invading lineage can also be interpreted as initial rounds of affinity maturation in germinal centers isolated from competition with adapted antibody lineages. Then the two antibody lineages are combined with resident at 90% and invader at 10%, with a total size of 10^3^, and the state of the system is recorded. The two lineages are evolved until one is extinct, repeated over 100 replicates to estimate the fixation probability. The whole procedure is repeated 10^3^ times for ensemble averaging. The invader, is either a normal lineage with all *κ*_*i*_ = 1 and ${\hat{\kappa }}_{i}=0$ or a BnAb that binds only to the conserved region, *κ*_*i*_ = 0 and ${\hat{\kappa }}_{i}=1$.

Simulations are written in julia and code is available at https://github.com/jotwin/coevolution.

### Analysis of HIV neutralization data

The data from Richman *et al.* [11] provides time-shifted measurements of viral neutralization by a patient’s circulating antibodies. We approximated time-shifted viral fitness as the log-ratio of neutralization titer (up to a constant *c*_0_) for plasma (antibodies) sampled at time *t* + *τ* against viruses sampled at time *t*, relative to the control titer of the same plasma against the neutralization-sensitive virus (NL43) [46], *F_*V*;*τ*_*(*t*) = −log (titer(*V_t_*, *A*_*t*+*τ*_)/titer(NL43, *A*_*t*+*τ*_)) + *c*_0_; titer is the reciprocal of antibody dilution where inhibition reaches 50% (IC_50_) [11]. Measuring neutralization efficacy relative to NL43 control virus is necessary to account for the increasing (non-stationary) antibody response during infection, shown in S8 Fig. Fig 4C shows the time-shifted relative mean fitness *F*_*V*;*τ*_(*t*) averaged over all time-points *t*, evaluated for two patients (TN-1 & TN-3), after linearly interpolating the raw data to produce equal time shifts (3 months for TN-1 and 6 months for TN-3). We fit the data to the analytical expression given by eqs. (S102, S103) in S1 Text, by minimizing the mean squared error after scanning over four composite evolutionary variables: (i) nucleotide diversity, which we infer to be equal for antibodies and viruses *θ*_*a*_ ≃ *θ*_*v*_ = *θ*, (ii) selection component of the fitness flux in the viral population $S_{v}^{2}M_{V,2}$, (iii) selection component of the transfer flux from antibodies to viruses, −*S*_*a*_ *S*_*v*_ *M*_*A*,2_(*N*_*v*_/*N*_*a*_), and (iv) the constant *c*_0_. Due to the functional form of time-shifted fitness given by eqs. (S102, S103) in S1 Text, brute force parameter scanning is necessary for a convergent solution. Further details of data analysis and estimates of the fitted variables are given in Section F of S1 Text.

## Supporting Information

S1 Text Supplementary information and detailed analytical derivations. **(A)** Antibody-viral coevolution in genotype space, **(B)** Antibody-viral coevolution in phenotype space, **(C)** Fitness flux and coevolutionary transfer flux, **(D)** Signature of coevolution from time-shifted fitness measurements, **(E)** Evolution of multiple antibody lineages, and **(F)** Analysis of time-shifted neutralization data.
(PDF)

S1 FigEffect of selection on the mean binding affinity.The rescaled mean binding affinity for **(A)** the variable interaction region $\varepsilon =E/E_{0}$, and **(B)** the conserved region $\hat{\varepsilon }=\hat{E}/E_{0}$, as a function of selection coefficients. Stationary mean binding affinity is sensitive to selection on antibodies in both variable and conserved regions. The conserved region is not sensitive to viral selection strength. Points indicate simulation results, dashed lines indicate the stationary solution given by eqs. (S59, S61) in S1 Text, using estimates for the diversity of the binding affinity from the simulations, and solid lines are the stationary solutions (S59, S61) in S1 Text using the analytical estimates of the diversity from eq. (S75) in S1 Text. Parameters are: $\kappa_{i}={\hat{\kappa }}_{i}=1$ for all sites, $\ell =\hat{\ell }=50$, *N*_*a*_ = *N*_*v*_ = 1000, *θ*_*a*_ = *θ*_*v*_ = 1/50. Points are time averaged values from simulations run for 10^6^ *N*_*a*_ generations, with values sampled every *N*_*a*_ generations, and data from first 100*N*_*a*_ generations discarded.(EPS)

S2 FigEffect of selection on the diversity and covarinace of binding affinity in antibodies and viruses.Stationary diversity of the binding affinity for **(A)** the variable interaction region $m_{A,2}=M_{A,2}/E_{0}^{2}$, **(B)** the conserved interaction region ${\hat{m}}_{A,2}={\hat{M}}_{A,2}/{\hat{E}}_{0}^{2}$ in the antibody population, and **(C)** the variable region in the viral population $m_{V,2}=M_{V,2}/E_{0}^{2}$ plotted as a function of viral and antibody selection coefficients. The diversity of binding across the antibodies in the conserved region ${\hat{m}}_{A,2}$ in (B) is not sensitive to viral selection strength. **(D)** The magnitude of the rescaled covariance due to genetic linkage between binding of the antibody to the conserved and the variable regions, $\langle {\left[\left(E_{\alpha \;.}-E\right)\left({\hat{E}}_{\alpha \;.}-\hat{E}\right)\right]}_{{}_{A}}\rangle /E_{0}{\hat{E}}_{0}$, is much smaller than the diversity of binding in each region, shown in (A) and (B). Points indicate simulation results with parameters similar to S1 Fig, dashed lines indicate the stationary solution using estimates for higher moments from the simulations (eqs. (S63, S64) in S1 Text), and solid lines indicate the full stationary solution given by eq. (S75) in S1 Text for antibodies, and the corresponding solution for viruses. Theory lines begin to deviate from simulation results for large selection strengths *s*_*a*_, *s*_*v*_ > 1. The deviations are larger in antibodies due to neglecting the linkage correlation between the variable and the conserved regions.(EPS)

S3 FigTime-dependent statistics.Auto-correlation of the stationary mean binding affinity in the variable region (red), eq. (S81) in S1 Text, has a shorter decay time than in the conserved region (yellow), eq. (S82) in S1 Text. The decay time for the auto-correlation of the trait mean in both variable and conserved regions, which is of order of the inverse mutation rate, is much longer than the correlation time of the second moments (green, blue, purple), which decay on a timescale of *N* generations. Solid lines are from stationary simulations, and dashed lines are the analytical results for the auto-covariance of the moments given by, eq. (S81) (red), eq. (S82) (yellow) and eq. (S83) (black) in S1 Text, normalized to have magnitude 1 at separation time Δ*τ* = 0. Parameters are: all $\kappa_{i}={\hat{\kappa }}_{i}=1$, $\ell =\hat{\ell }=50$, *N*_*a*_ = *N*_*v*_ = 1000, *θ*_*a*_ = *θ*_*v*_ = 1/50, $s_{a}=s_{v}={\hat{s}}_{a}=1$. Simulation results are time-averaged over 10^4^ *N*_*a*_ generations, with values sampled every *N*_*a*_ generations, and first 100*N*_*a*_ generations omitted.(EPS)

S4 FigAlternative fitness models.**(A)** Stationary mean binding affinity and **(B)** rate of antibody adaptation (fitness flux) due to selection, estimated by population fitness variance, for the nonlinear-averaged fitness model (black) and the nonlinear-EVD fitness model with the number of interactions, *R* = 10 (red), *R* = 100 (green), and *R* = 1000 (blue). The mean binding affinity is sensitive to the degree of non-linearity *β*, and binding threshold e*, but it is not very sensitive to the number of interactions *R*. The selection coefficient *s*_*a*_ is defined as in eq. (S39) in S1 Text. Dashed line in (B) indicates the expected fitness variance for a linear-averaged fitness model, $\langle \phi_{{}_{A}}\rangle ≃s_{a}^{2}\langle m_{A,2}\rangle$, which is the selection component of the fitness flux in eq. (S91) in S1 Text. Parameters are: $\kappa_{i}={\hat{\kappa }}_{i}=1$ for all sites, *ℓ* = 50, $\hat{\ell }=0$, *N*_*a*_ = *N*_*v*_ = 1000, *θ*_*a*_ = *θ*_*v*_ = 1/50. Points are time averaged values from simulations run for 10^5^ *N*_*a*_ generations, with values sampled every *N*_*a*_ generations, and data from first 100*N*_*a*_ generations discarded.(EPS)

S5 Fig Stationary time-shifted binding affinity between antigens and antibodies. Analytical estimates (dashed lines, eqs. (S102, S103) in S1 Text) for the ensemble-averaged time-shifted binding affinity 〈*ε*_*τ*_〉 between the viral population sampled at time *t*, and the antibody population at time *t* + *τ* averaged over all *t* in the stationary state, show good agreements with the numerical estimates of Wright-Fisher simulations (full lines), over a range of evolutionary parameters. Parameters are *N*_*a*_ = *N*_*v*_ = 1000 and *κ*_*i*_ = 1 for all sites, and the selection coefficients and the nucleotide diversity as indicated by the legend. Results are time-averaged over 10^4^ *N*_*a*_ generations, with first 100*N*_*a*_ generations omitted.
(EPS)

S6 FigNon-stationary signature of coevolution from time-shifted fitness.Transient (non-stationary) coevolution is quantified by the ensemble-averaged time-shifted mean fitness of the viral population sampled at a reference time point of *N*_*v*_ generations after the beginning of the simulation, that is before the system reaches a stationary state; see eqs. (S81, S82) in S1 Text. For *τ* > 0, the time-shifted fitness 〈*F*_*V*;*τ*_ (0)〉 (S99) measures the fitness of the focal viral population at reference time 0 against the antibodies sampled at time +*τ*. For *τ* < 0, we show 〈*F*_*V*;−*τ*_ (*τ*)〉, i.e., the time-shifted fitness with antibodies from *t* = 0 and viruses from time +*τ*. The fitness function is shown for two evolutionary regimes, (i) stronger viral selection, *s*_*v*_ = 2, *s*_*a*_ = 1 (red) and (ii) weaker viral selection, *s*_*v*_ = 1, *s*_*a*_ = 2 (blue). The slope of time-shifted fitness at *τ* = 0 measures the population’s fitness flux (dashed lines) and the transfer flux from the opposing population (dotted lines), estimated based on the phenotype statistics measured in the simulations (S92, S93). Fitness flux and transfer flux do not have equal values in a non-stationary state, leading to the discontinuity in the slope of the time-shifted fitness function at *τ* = 0. Parameters are $\ell =\hat{\ell }=50$, *N*_*a*_ = *N*_*v*_ = 1000, *θ*_*a*_ = *θ*_*v*_ = 1/50. Populations are evolved for *N*_*v*_ generations to reach the reference time *τ* = 0, then data is collected over 100*N*_*v*_ generations. Results are ensemble-averaged over 10^3^ initializations.(EPS)

S7 Fig Neutralization titers of HIV against patients plasma. Neutralization activity (titer) of plasma against autologous viruses collected at various time-points (colors) from two HIV patients, **(A)** TN-1 and **(B)** TN-3 as reported by [11]. Neutralization titers are defined as the reciprocal of antibody dilution at the level that inhibition reaches 50% (IC_50_). In addition, plasma activity against a neutralization-sensitive virus (NL43) is taken as a control measurement (dashed line), which indicates an increasing antibody response over time.
(EPS)
