# Supplementary material for: Host-Pathogen Coevolution and the Emergence of Broadly Neutralizing Antibodies in Chronic Infections
Source: PLoS Genet. 2016 Jul 21;12(7):e1006171. doi: 10.1371/journal.pgen.1006171 (PMC4956326; doi:10.1371/journal.pgen.1006171)
Supplement: S1 Text — (A) Antibody-viral coevolution in genotype space, (B) Antibody-viral coevolution in phenotype space, (C) Fitness flux and coevolutionary transfer flux, (D) Signature of coevolution from time-shifted fitness measurements, (E) Evolution of multiple antibody lineages, and (F) Analysis of time-shifted neutralization data. (PDF) [file pgen.1006171.s001.pdf]

S1 Text

A Antibody-viral coevolution in genotype space

We represent the antibody population as a set of k genotypes consisting of vectors, $\mathbf{A}^{\alpha}$ ($\alpha = 1 \dots k$), and corresponding genotype frequencies $\mathbf{x}$, with elements x^{α} satisfying $\sum_{\alpha=1}^{k} x^{\alpha} = 1$. Similarly, we consider a viral population with k' possible genotypes $\mathbf{V}^{a}$, and frequencies $\mathbf{y}$ with elements y^{γ} ($\gamma = 1, \dots, k'$) with $\sum_{\gamma=1}^{k'} y^{\gamma} = 1$. Note that superscripts are indices, not exponentiation, unless next to parentheses, e.g. $(a)^{b}$. The frequencies change over time, although we omit explicit notation for brevity, and hence every quantity that depends on the frequencies is itself time-dependent. In the following, we describe separately contributions from three evolutionary forces (i) mutation, (ii) selection, and (iii) genetic drift, and build a general stochastic framework for coevolution of antibodies and viruses in the space of genotypes. We assume that population sizes are large enough, and changes in frequencies are small enough to accommodate a continuous time and continuous frequency stochastic process [1, 2].

(i) Mutations. The change of the genotype frequencies due to mutations follow,

$$\frac{dx^{\alpha}}{dt} = m_{A^{\alpha}}(\mathbf{x}) \equiv \sum_{\beta=1}^{k} \mu_{A^{\beta}\to A^{\alpha}} x^{\beta} - \left(\sum_{\beta=1}^{k} \mu_{A^{\alpha}\to A^{\beta}} \right) x^{\alpha}$$
$$\frac{dy^{\gamma}}{dt} = m_{V^{\gamma}}(\mathbf{y}) \equiv \sum_{\lambda=1}^{k'} \mu_{V^{\lambda}\to V^{\gamma}} y^{\lambda} - \left(\sum_{\lambda=1}^{k'} \mu_{V^{\gamma}\to V^{\lambda}} \right) y^{\gamma} \tag{S1}$$

where we define $m_{A^{\alpha}}$ and $m_{V^{\gamma}}$ as the genotype-specific components of the mutational fields in the antibodies and viruses, and $\mu_{A^{\beta}\to A^{\alpha}}$ is the antibody mutation rate from genotype $\mathbf{A}^{\beta}$ to $\mathbf{A}^{\alpha}$, and similarly, $\mu_{V^{\lambda}\to V^{\gamma}}$ is the viral mutation rate from the genotype $\mathbf{V}^{\lambda}$ to $\mathbf{V}^{\gamma}$. We assume constant mutation rates μ_a, μ_v, per generation per site for antibodies and viruses, with the exception of $\mu_v = 0$ for the viral constant region, which implies that mutations in that region are lethal for the virus.

(ii) Selection and interacting fitness functions. The fitness of a genotype determines its growth rate at each point in time. We define fitness of genotypes in one population as a function of the genotypes in the other population. The general form of change in genotype frequencies due to selection follows,

$$\frac{1}{x^{\alpha}}\frac{dx^{\alpha}}{dt} = f_{A^{\alpha}}(\mathbf{x};\mathbf{y}) - \sum_{\alpha} x^{\alpha} f_{A^{\alpha}}(\mathbf{x};\mathbf{y})$$
$$\frac{1}{y^{\gamma}}\frac{dy^{\gamma}}{dt} = f_{V^{\gamma}}(\mathbf{y};\mathbf{x}) - \sum_{\gamma} y^{\gamma} f_{V^{\gamma}}(\mathbf{y};\mathbf{x}) \tag{S2}$$

The subscript for the antibody and viral fitness functions, $f_{A^{\alpha}}(\mathbf{x};\mathbf{y})$ and $f_{V^{\gamma}}(\mathbf{y};\mathbf{x})$, refer to the genotypes in the corresponding population. The explicit conditional dependence of the antibody fitness function $f_{A^{\alpha}}(\mathbf{x};\mathbf{y})$ on the viral frequency vector $\mathbf{y}$ emphasizes that fitness of an antibody depends on the interacting viral population $\{\mathbf{V}\}$. Similar notation is used for the fitness function of the viruses. The subtraction of the population's mean fitness, $F_{A} = \sum_{\alpha} x^{\alpha} f_{A^{\alpha}}(\mathbf{x};\mathbf{y})$ and $F_{V} = \sum_{\gamma} y^{\gamma} f_{V^{\gamma}}(\mathbf{y};\mathbf{x})$, ensures that the genotype frequencies remain normalized in each population. In terms of linearly independent frequencies $\mathbf{x} = (x^1, \dots, x^{k-1})$ and $\mathbf{y} = (y^1, \dots, y^{k'-1})$, these evolution equations take the forms,

$$\frac{dx^{\alpha}}{dt} = \sum_{\text{antibodies: } \beta} g^{\alpha\beta} \sigma_{A^{\beta}}(\mathbf{x};\mathbf{y}), \qquad \frac{dy^{\gamma}}{dt} = \sum_{\text{viruses: } \lambda} h^{\gamma\lambda} \sigma_{V^{\lambda}}(\mathbf{y};\mathbf{x}) \tag{S3}$$

where $\sigma_{A^\alpha}(\mathbf{x};\mathbf{y}) = f_{A^\alpha}(\mathbf{x};\mathbf{y}) - f_{A^k}(\mathbf{x};\mathbf{y})$ and $\sigma_{V^\gamma}(\mathbf{y};\mathbf{x}) = f_{V^\gamma}(\mathbf{y};\mathbf{x}) - f_{V^{k'}}(\mathbf{y};\mathbf{x})$ are the respective time-dependent selection coefficients of the antibody $\mathbf{A}^\alpha$ and the viral strain $\mathbf{V}^\gamma$, which depend on the state of the both populations at that moment in time. The inverse of the response matrices, $g_{\alpha\beta} = (g^{\alpha\beta})^{-1}$ and $h_{\gamma\lambda} = (h^{\gamma\lambda})^{-1}$, play the role of metric in the genotype space (see below and e.g., [3]). The change in the mean fitness due to selection after an infinitesimal amount of time follows,

$$F_A(\mathbf{x}+\delta\mathbf{x};\mathbf{y}+\delta\mathbf{y}) = \sum_\alpha \sigma_{A^\alpha}(\mathbf{x};\mathbf{y})\delta x^\alpha + \sum_{\gamma,\alpha} x^\alpha\, \sigma_{V^\gamma \to A^\alpha}(\mathbf{x};\mathbf{y})\,\delta y^\gamma \tag{S4}$$

$$F_V(\mathbf{y}+\delta\mathbf{y};\mathbf{x}+\delta\mathbf{x}) = \sum_\gamma \sigma_{V^\gamma}(\mathbf{y};\mathbf{x})\delta y^\gamma + \sum_{\gamma,\alpha} y^\gamma\, \sigma_{A^\alpha \to V^\gamma}(\mathbf{y};\mathbf{x})\,\delta x^\alpha \tag{S5}$$

where δx^α and δy^γ are the infinitesimal changes in the genotype frequencies, and $\sigma_{V^\gamma \to A^\alpha} = \partial\sigma_{A^\alpha}/\partial y^\gamma$ and, $\sigma_{A^\alpha \to V^\gamma} = \partial\sigma_{V^\gamma}/\partial x^\alpha$ are respectively the change in the selection coefficient of the antibody $\mathbf{A}^\alpha$ and the virus $\mathbf{V}^\gamma$ due the evolution of opposing population. This measure of fitness transfer is a useful concept for interacting populations. Intuitively, it can be understood as the change of fitness in one population only due to the changes of allele or genotype frequencies in the opposing population.

(iii) Genetic drift and stochasticity. The stochasticity of reproduction and survival, commonly known as genetic drift, is represented as discrete random sampling of offspring genotypes from the parent's generation with the constraint that the total population size remains constant. The magnitude of this sampling noise is proportional to inverse population size. N_a and N_v are the effective population sizes of the antibody and the viral populations, which represent the size of population bottlenecks e.g., in a germinal center. In the continuous time, continuous frequency limit, genetic drift is represented as noise terms in a diffusion equaiton with magnitude proportional to inverse population size [2]. The diffusion coefficients are characteristics of the Fisher metric [4, 3],

$$g^{\alpha\beta} = \begin{cases} -x^\alpha x^\beta & \text{if } \alpha \neq \beta \\ x^\alpha(1-x^\alpha) & \text{if } \alpha = \beta \end{cases}, \qquad h^{\gamma\lambda} = \begin{cases} -y^\gamma y^\lambda & \text{if } \gamma \neq \lambda \\ y^\gamma(1-y^\gamma) & \text{if } \gamma = \lambda \end{cases} \tag{S6}$$

The generalized Kimura's diffusion equation [5] for the joint distribution of genotype frequencies $P(\mathbf{x},\mathbf{y},t)$ in both populations reads,

$$\begin{aligned}\frac{\partial}{\partial t}P(\mathbf{x},\mathbf{y},t) = \sum_{\alpha,\beta,\gamma,\lambda} \Big[&\frac{1}{2N_a}\frac{\partial^2}{\partial x^\alpha x^\beta} g^{\alpha\beta}(\mathbf{x}) + \frac{1}{2N_v}\frac{\partial^2}{\partial y^\gamma \partial y^\lambda} h^{\gamma\lambda}(\mathbf{y}) \\ &+ \frac{\partial}{\partial x^\alpha}\big(m_A^\alpha(\mathbf{x}) + g^{\alpha\beta}(\mathbf{x})\,\sigma_{A^\beta}(\mathbf{x};\mathbf{y})\big) + \frac{\partial}{\partial y^\gamma}\big(m_V^\gamma(\mathbf{y}) + h^{\gamma\lambda}(\mathbf{y})\sigma_{V^\lambda}(\mathbf{y};\mathbf{x})\big)\Big] P(\mathbf{x},\mathbf{y},t)\end{aligned} \tag{S7}$$

This Fokker-Planck equation acts on the high-dimensional genotype space of antibodies and viruses, which are likely to be under-sampled in any biological setting. In the following, we introduce a projection from genotype space onto a lower dimensional space of molecular traits (phenotypes) to make the problem tractable.

B Antibody-viral coevolution in phenotype space

B.1 Molecular phenotypes for antibody-viral interaction

We define the binding affinity between an antibody and viral genotype as the molecular interaction phenotype under selection, for which we describe the evolutionary dynamics. Antibody and viral genotypes are represented by binary sequences of ± 1. Antibody sequences are of length $\ell + \hat{\ell}$, while viral sequences consist of a mutable region of length ℓ, and a conserved (i.e. sensitive) region of length $\hat{\ell}$, where each site is always $+1$, as was similarly done in [6]. We model the binding affinity between antibody $\mathbf{A}^\alpha$ and virus $\mathbf{V}^\gamma$ as a weighted dot product over all sites,

$$E_{\text{tot}}(\mathbf{A}^\alpha, \mathbf{V}^\gamma) = \sum_{i=1}^{\ell} \kappa_i A_i^\alpha V_i^\gamma + \sum_{i=\ell+1}^{\ell+\hat{\ell}} \hat{\kappa}_i \, A_i^\alpha$$
$$\equiv E_{\alpha\,\gamma} + \hat{E}_\alpha \tag{S8}$$

where A_i^α, and V_i^γ denote the ith site in antibody $\mathbf{A}^\alpha$ and virus $\mathbf{V}^\gamma$, respectively. The set of coupling constants for the mutable and conserved region, $\{\kappa_i, \hat{\kappa}_i \geq 0\}$ represent the accessibility of a clonal antibody lineage to regions of the viral sequence. Matching bits at interacting positions enhance binding affinity between an antibody and a virus. Similar models have been used to describe B-cell maturation in germinal centers [6], and T-cell selection based on the capability to bind external antigens and avoid self proteins [7, 8]. In Section E, we extend our model to multiple lineages, where each lineage has its own set of accessibilities. Antibody lineages with access to the conserved regions of the virus can potentially fix as broadly neutralizing antibodies. We denote the quantities related to the conserved sites of the virus with a hat: $\hat{\cdot}$.

We project the evolutionary forces acting on the genotype to the binding phenotype E_{tot}, and quantify the changes of the binding phenotype distribution in each population over time. For a single antibody genotype $\mathbf{A}^\alpha$ we characterize its interactions with the viral population by the *genotype-specific moments*,

Statistics of the binding affinity distribution for antibody $\mathbf{A}^\alpha$:

(i) average in the variable region:
$$E_{\alpha\,.} = \sum_{\gamma \in \text{ viruses}} E_{\alpha\,\gamma} y^\gamma \tag{S9}$$
(ii) average in the conserved region:
$$\hat{E}_{\alpha\,.} = \hat{E}_\alpha \tag{S10}$$
(iii) r^{th} central moment in the variable region:
$$I_{\alpha\,.}^{(r)} = \sum_{\gamma \in \text{ viruses}} (E_{\alpha\,\gamma} - E_{\alpha\,.})^r y^\gamma \tag{S11}$$

Since the viral population is monomorphic in the conserved region, the average mean binding affinity of an antibody is independent of the state of the viral population, $\hat{E}_{\alpha\,.} = \hat{E}_\alpha$, and the higher central moments are zero, $\hat{I}_{\alpha\,.}^{(r)} = 0$. Similarly, we characterize the interactions of a given viral genotype $\mathbf{V}^\gamma$ with the antibody population,

Statistics of the binding affinity distribution for virus $\mathbf{V}^\gamma$:

(i) average in the variable region:
$$E_{.\,\gamma} = \sum_{\alpha \in \text{antibodies}} E_{\alpha\,\gamma} x^\alpha \tag{S12}$$
(ii) average in the conserved region:
$$\hat{E}_{.\,\gamma} = \sum_{\alpha \in \text{antibodies}} \hat{E}_\alpha x^\alpha \equiv \hat{E}_. \tag{S13}$$
(iii) r^{th} central moment in the variable region:
$$I_{.\,\gamma}^{(r)} = \sum_{\alpha \in \text{antibodies}} (E_{\alpha\,\gamma} - E_{.\,\gamma})^r x^\alpha \tag{S14}$$
(iii) r^{th} central moment in the conserved region:
$$\hat{I}_{.\,\gamma}^{(r)} = \sum_{\alpha \in \text{antibodies}} (\hat{E}_\alpha - \hat{\mathcal{E}})^r x^\alpha \tag{S15}$$

One of the most informative statistics that we characterize is the distribution of population-averaged antibody and viral binding interactions, respectively denoted by $P_A(E_{\alpha\,.}, \hat{E}_\alpha)$ and $P_V(E_{.\,\gamma}, \hat{E}_.)$. The mean of these distributions

are equal to each other, but the higher moments differ. We denote the *population-specific moments* of the average interactions by,

Mean binding affinity in,

$$\text{(i) the variable region:} \qquad \mathcal{E} = \sum_{\alpha,\gamma} E_{\alpha\,\gamma}\, x^{\alpha} y^{\gamma} \tag{S16}$$

$$\text{(ii) the conserved region:} \qquad \hat{\mathcal{E}} = \sum_{\alpha} \hat{E}_{\alpha}\, x^{\alpha} \tag{S17}$$

r^{th} **central moment of the average affinities in,**

(i) the variable region of antibody population:

$$M_{A,r} = \sum_{\alpha \in \text{antibodies}} (E_{\alpha\,\cdot} - \mathcal{E})^r x^{\alpha} \tag{S18}$$

(ii) the conserved region of antibody population:

$$\hat{M}_{A,r} = \sum_{\alpha \in \text{antibodies}} (\hat{E}_{\alpha\,\cdot} - \hat{\mathcal{E}})^r x^{\alpha} \tag{S19}$$

(iii) the variable region of viral population:

$$M_{V,r} = \sum_{\gamma \in \text{viruses}} (E_{\cdot\,\gamma} - \mathcal{E})^r y^{\gamma} \tag{S20}$$

Note that the population central moments $M_{A,r}$ and $M_{V,r}$ are distinct from the genotype-specific moments, $I^{(r)}_{\alpha\,\cdot}$ and $I^{(r)}_{\cdot\,\gamma}$. The central moments of the viral population in the conserved region are equal to zero, $\hat{M}_{V,r} = 0$.

Trait scale and dimensionless quantities. It is useful to measure traits in natural units, which avoids the arbitrariness of the physical units $(\{\kappa_i,\, \hat{\kappa}_i\})$, and the total number of sites $\ell + \hat{\ell}$. As previously shown in [9, 10], there exist summary statistics of the site-specific effects, (here $\{\kappa_i, \hat{\kappa}_i\}$), which define a natural scale of the molecular phenotype. We denote the moments of the site-specific effects along the genome by,

$$\mathcal{K}_r = \frac{1}{\ell} \sum_{i=1}^{\ell} (\kappa_i)^r, \qquad \hat{\mathcal{K}}_r = \frac{1}{\hat{\ell}} \sum_{i=\ell+1}^{\ell+\hat{\ell}} (\hat{\kappa}_i)^r \tag{S21}$$

We express the phenotype statistics in units of the trait scales, i.e., the squared sum of the site-specific effects, $E_0^2 = \mathcal{K}_2 \ell$ in the variable region, and $\hat{E}_0^2 = \hat{\mathcal{K}}_2 \hat{\ell}$ in the conserved region. The rescaled phenotype statistics follow,

$$\varepsilon \equiv \frac{\mathcal{E}}{E_0}, \qquad \hat{\varepsilon} \equiv \frac{\hat{\mathcal{E}}}{\hat{E}_0} \qquad \text{and,} \qquad m_{Z,r} \equiv \frac{M_{Z,r}}{E_0^r}, \qquad \hat{m}_{Z,r} \equiv \frac{\hat{M}_{Z,r}}{\hat{E}_0^r} \qquad (\text{for } Z = A, V) \tag{S22}$$

These scaled values are pure numbers (we distinguish them by use of lower case letters from the raw data). The trait scales E_0^2 and $\hat{E}_0^2$ provide natural means to standardize the relevant quantities because they are the stationary ensemble variances of the population mean binding affinity in an ensemble of genotypes undergoing neutral evolution in the weak-mutation regime (see Section B.3 for derivation of the stationary statistics),

$$E_0^2 = \lim_{\mu_v, \mu_a \to 0} \langle (\mathcal{E} - \langle \mathcal{E} \rangle)^2 \rangle, \qquad \hat{E}_0^2 = \lim_{\mu_a \to 0} \langle (\hat{\mathcal{E}} - \langle \hat{\mathcal{E}} \rangle)^2 \rangle \tag{S23}$$

where $\langle \cdot \rangle$ indicates averages over an ensemble of independent populations.

Binding probability. The probability that an antibody is bound by an antigen determines its chance of proliferation and survival during the process of affinity maturation, and hence, defines its fitness. We describe two distinct models

for antibody activation. The simplest model assumes that the binding probability of a given antibody $\mathbf{A}^\alpha$ is a sigmoid function of its *mean binding affinity* against the viral population,

$$p_{_A}(\mathbf{A}^\alpha) = \frac{1}{1+\exp[-\beta_0(E_{\alpha\,.} + \hat{E}_{\alpha\,.} - E^*)]} \tag{S24}$$

where E^* is the threshold for the binding affinity and β_0 determines the amount of nonlinearity, and is related to the inverse of temperature in thermodynamics. Following the rescaling introduced in eq. (S22), the binding threshold and the nonlinearity in eq. (S24) rescale as $e^* \equiv E^*/\sqrt{\hat{E}_0^2 + E_0^2}$ and $\beta = \beta_0\sqrt{\hat{E}_0^2 + E_0^2}$. In the following, we will use eq. (S24) to characterize a biophysically grounded fitness function for antibodies.

For the virus, binding to an antibody reduces the chances of its survival. Similar to eq. (S24), the probability that a given virus $\mathbf{V}^\gamma$ is bound by antibodies follows,

$$p_{_V}(\mathbf{V}^\gamma) = \frac{1}{1+\exp[-\beta_0(E_{.\,\gamma} + \hat{E}_{.\,\gamma} - E^*)]}, \tag{S25}$$

where E^* and β_0 are similar to eq. (S24).

In Section B.5, we will discuss an alternative model for activation of an antibody which is based on its *strongest binding affinity* with a subset of viruses.

B.2 Coevolutionary forces on the binding affinity

Similar to genotype evolution, stochastic evolution of a molecular phenotype generates a probability distribution, $Q(\mathcal{E}, \hat{\mathcal{E}}, M_{A,r}, \hat{M}_{A,r}, M_{V,r})$, which describes an ensemble of independently evolving populations, each having a phenotype distribution with mean affinity $\mathcal{E}$ and $\hat{\mathcal{E}}$ and central moments of the averaged affinity in the antibody population, $M_{A,r}$, $\hat{M}_{A,r}$, and in the viral population, $M_{V,r}$ (see also [9]). The probability distribution $Q(\mathcal{E}, \hat{\mathcal{E}}, M_{A,r}, \hat{M}_{A,r}, M_{V,r})$ can be expressed in therms of the distribution for genotype frequencies,

$$Q(\mathcal{E}, \hat{\mathcal{E}}, M_{A,r}, \hat{M}_{A,r}, M_{V,r}) = \int d\mathbf{x}d\mathbf{y}\, P(\mathbf{x},\mathbf{y},t)\left[\delta(\mathcal{E}(\mathbf{x},\mathbf{y}) - \mathcal{E})\,\delta(\hat{\mathcal{E}}(\mathbf{x}) - \hat{\mathcal{E}})\prod_r \delta(M_{A,r}(\mathbf{x},\mathbf{y}) - M_{A,r})\,\delta(\hat{M}_{A,r}(\mathbf{x}) - \hat{M}_{A,r})\,\delta(M_{V,r}(\mathbf{x},\mathbf{y}) - M_{V,r})\right] \tag{S26}$$

where $\delta(\cdot)$ is the Dirac delta function. Below, we characterize the effect of mutations, selection and genetic drift on the evolution of the phenotype moments $\mathcal{E}$, $M_{A,r}$, $\hat{M}_{A,r}$ and $M_{V,r}$.

Mutation. A mutation at site "i" changes the sign of the site, and its effect on the binding affinity is proportional to κ_i in the variable region, and $\hat{\kappa}_i$ in the conserved region. To compute the effect of mutations on moments of the phenotype distribution, we classify pairs of genotypes $(\mathbf{A}^\alpha, \mathbf{V}^\gamma)$ in mutational classes, defined by the number of $+1$ positions of their product vector $(A_1^\alpha \cdot V_1^\gamma, \dots, A_{\ell+\hat{\ell}}^\alpha \cdot V_{\ell+\hat{\ell}}^\gamma)$, which we denote by n_+ in the variable region and by $\hat{n}_+$ in the conserved interaction region,

$$n_+(\mathbf{A}^\alpha, \mathbf{V}^\gamma) = \sum_{i=1}^{\ell} \delta(1 - A_i^\alpha \cdot V_i^\gamma), \qquad \hat{n}_+(\mathbf{A}^\alpha, \mathbf{V}^\gamma) = \sum_{i=\ell+1}^{\ell+\hat{\ell}} \delta(1 - A_i^\alpha \cdot V_i^\gamma) \tag{S27}$$

The frequency of each mutational class $\mathcal{Q}(n_+)$ is estimated from interactions between all pairs of antibody and viral genotypes in both variable and conserved regions of the interacting populations,

$$\mathcal{Q}^{(1)}(n_+) = \frac{1}{N_a N_V}\sum_{\alpha,\gamma} \delta(n_+(\mathbf{A}^\alpha, \mathbf{V}^\gamma) - n_+), \qquad \mathcal{Q}^{(2)}(n_+) = \frac{1}{N_a N_V}\sum_{\alpha,\gamma} \delta(\hat{n}_+(\mathbf{A}^\alpha, \mathbf{V}^\gamma) - \hat{n}_+) \tag{S28}$$

The superscript $\lambda = 1, 2$ indicates the interacting region of the virus, i.e. $\lambda = 1$ refers to the variable region of the virus with $\mu_v^{(1)} = \mu_v$ and the length $\ell^{(1)} = \ell$, and $\lambda = 2$ refers to the conserved region of the viral genome with $\mu_v^{(2)} = 0$ and the sequence length $\ell^{(2)} = \hat{\ell}$. If the mutational effects of all sites were equal to κ, phenotype moments could be simply expressed using the statistics of mutational classes: e.g., $\mathcal{E} = (2[n_+]_{A,V} - \ell)\kappa$, where $[\cdot]_{A,V}$ indicates averaging of a quantity in the subscript populations, which in this case are both the viral and the antibody populations. If the number of encoding sites of a phenotype is large, *annealed averages* of the heterogeneous site-specific contributions $\mathcal{K}_r$, $\hat{\mathcal{K}}_r$ can well approximate the the moments of the phenotype distribution [11, 12, 9]. As a result, the statistics of the variable region follow, $\mathcal{E} = (2[n_+]_{A,V} - \ell)\mathcal{K}_1$ for the mean binding affinity, and $M_{V,r} = 2^r \mathcal{K}_r \Big[\big([n_+]_A - [n_+]_{A,V}\big)^r \Big]_V$, $M_{A,r} = 2^r \mathcal{K}_r \Big[\big([n_+]_V - [n_+]_{A,V}\big)^r \Big]_A$ for the higher central moments in viruses and antibodies. Similar expressions can be derived for the statistics of the conserved region. Therefore, evolution of the phenotype distribution can be well-approximated using projections from evolutionary dynamics of the mutational classes. The Master equation for the evolution of the mutational classes under neutrality (mutation and genetic drift) follows,

$$\begin{aligned} d\,\mathcal{Q}^{(\lambda)}(n_+) &= (\mu_a + \mu_v^{(\lambda)}) \Big[(\ell^{(\lambda)} - (n_+ - 1))\mathcal{Q}^{(\lambda)}(n_+ - 1) + (n_+ + 1)\mathcal{Q}^{(\lambda)}(n_+ + 1) - \ell^{(\lambda)}\,\mathcal{Q}^{(\lambda)}(n_+) \Big] dt \\ &\quad + \big(\delta_{n'_+, n_+} - \mathcal{Q}^{(\lambda)}(n_+)\big) \left[\sqrt{\frac{\mathcal{Q}^{(\lambda)}(n_+)}{N_a}} dW_A(t) + \sqrt{\frac{\mathcal{Q}^{(\lambda)}(n_+)}{N_v}} dW_V(t) \right] \end{aligned} \quad \text{(S29)}$$

$W_A(t)$ and $W_V(t)$ are delta-correlated Gaussian noise (Wiener process) with an ensemble mean $\langle W_i \rangle = 0$ and variance, $\langle W_i(t) W_j(t') \rangle = \delta_{i,j}\,\delta(t - t')$ where $i, j \in \{A, V\}$ indicate antibodies and viruses. The stochasticity (genetic drift) is due to finite population size of the interacting genotypes N_a and N_v.

In neutrality, the ensemble mean for the averaged number of positive sites $\big\langle [n_+{}^{(\lambda)}]_{A,V} \big\rangle$ and the central moments, $\langle Y_{A,r}^{(\lambda)} \rangle \equiv \Big\langle \big[\big([n_+^{(\lambda)}]_V - [n_+^{(\lambda)}]_{A,V}\big)^r \big]_A \Big\rangle$ and $\langle Y_{V,r}^{(\lambda)} \rangle \equiv \Big\langle \big[\big([n_+^{(\lambda)}]_A - [n_+^{(\lambda)}]_{A,V}\big)^r \big]_V \Big\rangle$ in both variable ($\lambda = 1$) and conserved ($\lambda = 2$) interaction regions follow [11, 12],

$$\begin{aligned} \frac{\partial \langle [n_+{}^{(\lambda)}]_{A,V} \rangle}{\partial t} &= \left\langle (\mu_a + \mu_v^{(\lambda)}) \sum_{n_+} n_+ \Big[(\ell^{(\lambda)} - n_+ + 1)\mathcal{Q}^{(\lambda)}(n_+ - 1) + (n_+ + 1)\mathcal{Q}^{(\lambda)}(n_+ + 1) - \ell^{(\lambda)}\mathcal{Q}^{(\lambda)}(n_+) \Big] \right\rangle \\ &= \begin{cases} -2(\mu_a + \mu_v) \Big[(\langle [n_+]_{A,V} \rangle - \ell/2 \Big) & \text{variable region}, \lambda = 1 \\ -2\mu_a \Big(\big\langle [n_+]_{A,V} \big\rangle - \hat{\ell}/2 \Big) & \text{constant region}, \lambda = 2 \end{cases} \end{aligned} \quad \text{(S30)}$$

$$\begin{aligned} \frac{\partial}{\partial t} \left\langle Y_{A,r}^{(\lambda)} \right\rangle &= \mu_a \ell^{(\lambda)} \sum_{i=0}^{r-2} \binom{r}{i} \left\langle Y_{A,i}^{(\lambda)} \right\rangle + \frac{\binom{r}{2} \left\langle Y_{A,2}^{(\lambda)} Y_{A,r-2}^{(\lambda)} \right\rangle - r \left\langle Y_{A,r}^{(\lambda)} \right\rangle}{N_a} - 2r(\mu_a + \mu_v^{(\lambda)}) \left\langle Y_{A,r}^{(\lambda)} \right\rangle \\ &\quad - \mu_a \sum_{i=0}^{r-2} \binom{r}{i} \left(\left\langle Y_{A,i+1}^{(\lambda)} \right\rangle + \left\langle [n_+]_{A,V} Y_{A,i}^{(\lambda)} \right\rangle \right) [1 + (-1)^{r-i+1}] \end{aligned} \quad \text{(S31)}$$

$$\begin{aligned} \frac{\partial}{\partial t} \left\langle Y_{V,r}^{(\lambda)} \right\rangle &= \mu_v^{(\lambda)} \ell^{(\lambda)} \sum_{i=0}^{r-2} \binom{r}{i} \left\langle Y_{V,i}^{(\lambda)} \right\rangle + \frac{\binom{r}{2} \left\langle Y_{V,2}^{(\lambda)} Y_{V,r-2}^{(\lambda)} \right\rangle - r \left\langle Y_{V,r}^{(\lambda)} \right\rangle}{N_v} - 2r(\mu_a + \mu_v^{(\lambda)}) \langle Y_{V,r}^{(\lambda)} \rangle \\ &\quad - \mu_v^{(\lambda)} \sum_{i=0}^{r-2} \binom{r}{i} \left(\left\langle Y_{V,i+1}^{(\lambda)} \right\rangle + \left\langle [n_+]_{A,V} Y_{V,i}^{(\lambda)} \right\rangle \right) [1 + (-1)^{r-i+1}] \end{aligned} \quad \text{(S32)}$$

where $\langle \cdot \rangle$ denotes averages over independent ensembles of populations. The second term in the right-hand side of equations (S31, S32) is a consequence of the Itô calculus in stochastic processes [1]. The transformations from $[n_+^{(1)}]_{A,V}$ to $\mathcal{E}$ in the variable region, and from $[n_+^{(2)}]_{A,V}$ to $\hat{\mathcal{E}}$ in the conserved region result in,

$$\frac{\partial\langle\mathcal{E}\rangle}{\partial t} = -2(\mu_a+\mu_v)\langle\mathcal{E}\rangle \tag{S33}$$

$$\frac{\partial\langle\hat{\mathcal{E}}\rangle}{\partial t} = -2\mu_a\langle\hat{\mathcal{E}}\rangle \tag{S34}$$

The transformations from $Y^{(1)}_{A,r}$ to $M_{A,r}$, from $Y^{(2)}_{A,r}$ to $\hat{M}_{A,r}$ and from $Y^{(1)}_{V,r}$ to $M_{V,r}$ result in,

$$\begin{aligned}\frac{\partial\langle M_{A,r}\rangle}{\partial t} &= \mu_a\ell\sum_{i=0}^{r-2}2^{r-i}\mathcal{K}_{r-i}\binom{r}{i}\langle M_{A,i}\rangle+\frac{\binom{r}{2}\mathcal{K}_2\,\mathcal{K}_{r-2}\langle M_{A,2}M_{A,r-2}\rangle-r\langle M_{A,r}\rangle}{N_a}-2r(\mu_a+\mu_v)\langle M_{A,r}\rangle\\&-\mu_a\sum_{i=0}^{r-2}2^{r-i-1}\binom{r}{i}\left[\mathcal{K}_{r-i-1}\langle M_{A,i+1}\rangle+\frac{\mathcal{K}_{r-i}}{\mathcal{K}_1}\langle\mathcal{E}M_{A,i}\rangle\right]\left[1+(-1)^{r-i+1}\right]\end{aligned} \tag{S35}$$

$$\begin{aligned}\frac{\partial\langle\hat{M}_{A,r}\rangle}{\partial t} &= \mu_a\hat{\ell}\sum_{i=0}^{r-2}2^{r-i}\mathcal{K}_{r-i}\binom{r}{i}\langle\hat{M}_{A,i}\rangle+\frac{\binom{r}{2}\mathcal{K}_2\,\mathcal{K}_{r-2}\langle\hat{M}_{A,2}\hat{M}_{A,r-2}\rangle-r\langle\hat{M}_{A,r}\rangle}{N_a}-2r\mu_a\langle\hat{M}_{A,r}\rangle\\&-\mu_a\sum_{i=0}^{r-2}2^{r-i-1}\binom{r}{i}\left[\mathcal{K}_{r-i-1}\langle\hat{M}_{A,i+1}\rangle+\frac{\mathcal{K}_{r-i}}{\mathcal{K}_1}\langle\hat{\mathcal{E}}\hat{M}_{A,i}\rangle\right]\left[1+(-1)^{r-i+1}\right]\end{aligned} \tag{S36}$$

$$\begin{aligned}\frac{\partial\langle M_{V,r}\rangle}{\partial t} &= \mu_v\ell\sum_{i=0}^{r-2}2^{r-i}\mathcal{K}_{r-i}\binom{r}{i}\langle M_{V,i}\rangle+\frac{\binom{r}{2}\mathcal{K}_2\,\mathcal{K}_{r-2}\langle M_{V,2}M_{V,r-2}\rangle-r\langle M_{V,r}\rangle}{N_v}-2r(\mu_v+\mu_a)\langle M_{V,r}\rangle\\&-\mu_v\sum_{i=0}^{r-2}2^{r-i-1}\binom{r}{i}\left[\mathcal{K}_{r-i-1}\langle M_{V,i+1}\rangle+\frac{\mathcal{K}_{r-i}}{\mathcal{K}_1}\langle\mathcal{E}M_{V,i}\rangle\right]\left[1+(-1)^{r-i+1}\right]\end{aligned} \tag{S37}$$

Selection. We assume that (malthusian) fitness of an antibody is proportional to the logarithm of its activation probability given by eq. (S24) based on its average interaction strength,

$$f_{A^\alpha}\equiv f_A(\mathbf{A}^\alpha;\{V\}) = c_a\log[p_A(\mathbf{A}^\alpha)] = -c_a\log(1+\exp[-\beta_0(E_{\alpha\cdot}+\hat{E}_{\alpha\cdot}-E^*)]) \tag{S38}$$

$$\simeq f^*_A+S_a(E_{\alpha\cdot}+\hat{E}_{\alpha\cdot}) \tag{S39}$$

with $f^*_A=-c_a\log\left(1+\exp[\beta_0E^*]\right)$ and the selection coefficient $S_a=c_a\beta_0/(1+\exp[-\beta_0E^*])$. The approximation in (S39) is by expansion of the nonlinear fitness function around the neutral binding affinity, $\mathcal{E}=0$. The antibody selection coefficient S_a can be thought as the amount of stimulation that a bound B-cell receptor experiences, e.g. due to helper T-cells. If the chronic infection is HIV, where the virus attacks the helper T-cells, S_a may decrease as HIV progresses and the T-cell count decays. Furthermore, f^*_A affects the absolute growth rate, but does not affect the relative growth rate between genotypes. We call the fitness models based on the averaged binding affinity in eq. (S38) as *nonlinear-averaged* and in eq. (S39) as *linear-averaged.* In Section B.5 we introduce an alternative model of antibody activation, which assumes that proliferation of an antibody is related to its *best binding affinity* against $R\leq N_v$ antigens, that are presented to the antibody during its life time. The analytical results in this paper are all based on the antibody evolution in linear-averaged fitness landscapes (S39), and the other fitness models are only studied numerically.

The viral fitness is related to the probability that it escapes the binding interactions with antibodies. We define the fitness of an antigen (virus) as the negative logarithm of its binding probability to the average antibodies that it interacts with, given by eq. (S25),

$$f_{V^\gamma} \equiv f_V(\mathbf{V}^\gamma; \{A\}) \quad = \quad -c_v \log[p_V(\mathbf{V}^\gamma)] = c_v \log(1 + \exp[-\beta_0(E_{.\gamma} + \hat{E}_{.\gamma} - E^*)]) \tag{S40}$$

$$\simeq \quad f_V^* - S_v(E_{.\gamma} + \hat{E}_{.\gamma}) \tag{S41}$$

with $f_V^* = c_v \log\left(1 + \exp[\beta_0 E^*]\right)$ and the selection coefficient $S_v = c_v\beta_0/(1 + \exp[-\beta_0 E^*])$.

As shown in eq. (S3) the change in the frequency of an antibody or a virus is proportional to its fitness, which is related to its average binding affinity. Therefore, the change of a given phenotype statistic $U(\mathbf{x}, \mathbf{y})$ due to selection follows,

$$\frac{d}{dt}U(\mathbf{x}, \mathbf{y}) = \sum_{\alpha,\gamma} \left[\frac{\partial U}{\partial x^\alpha}(f_{A^\alpha} - F_A)\, x^\alpha + \frac{\partial U}{\partial y^\gamma}(f_{V^\gamma} - F_V)\, y^\gamma\right] \tag{S42}$$

where F_A and F_V are respectively the mean fitness in the antibody and in the viral population. With this formulation we can compute the effect of selection on the statistics of the binding affinity distribution, i.e., the mean affinity $\mathcal{E}$, $\hat{\mathcal{E}}$, and the central moments, $M_{A,r}$, $\hat{M}_{A,r}$ and $M_{V,r}$, which we present in the following section.

Similar to the rescaling procedure in eq. (S22), we use the total trait scales to define the rescaled strength of selection,

$$s_a = N_a S_a E_0, \qquad \hat{s}_a = N_a S_a \hat{E}_0, \qquad s_v = N_a S_v E_0, \qquad \hat{s}_v = N_v S_v \hat{E}_0 \tag{S43}$$

Genetic drift. We can project the stochasticity of the genotype space onto the phenotype space. The projected diffusion coefficients show the correlation between the noise levels of the phenotypic statistics A and B.

$$\mathcal{G}^{AB} = \frac{1}{N_a}\sum_{\alpha,\beta} \frac{\partial A}{\partial x^\alpha}\,\frac{\partial B}{\partial x^\beta}\, g^{\alpha\beta} + \frac{1}{N_v}\sum_{\gamma,\lambda} \frac{\partial A}{\partial y^\gamma}\,\frac{\partial B}{\partial y^\lambda}\, h^{\gamma\lambda} \tag{S44}$$

and the genotypic diffusion constants $g^{\alpha\beta}$ and $h^{\gamma\lambda}$ are given by eq. (S6). As an example, we compute the diffusion term for the mean binding affinity in the variable region $\mathcal{E}$,

$$\begin{aligned}
\mathcal{G}^{\mathcal{E}\mathcal{E}} &= \frac{1}{N_a}\sum_{\alpha,\beta} \frac{\partial \mathcal{E}}{\partial x^\alpha}\,\frac{\partial \mathcal{E}}{\partial x^\beta}\, g^{\alpha\beta} + \frac{1}{N_v}\sum_{\gamma,\lambda} \frac{\partial \mathcal{E}}{\partial y^\gamma}\,\frac{\partial \mathcal{E}}{\partial y^\lambda}\, h^{\gamma\lambda} \\
&= \frac{1}{N_a}\sum_{\alpha,\beta} E_{\alpha.}E_{\beta.}\Big[-x^\alpha x^\beta(1-\delta_{\alpha\beta}) + x^\alpha(1-x^\alpha)\delta_{\alpha\beta}\Big] \\
&\quad + \frac{1}{N_v}\sum_{\gamma,\lambda} E_{.\gamma}E_{.\lambda}\Big[-y^\gamma y^\lambda(1-\delta_\gamma^\lambda) + y^\gamma(1-y^\gamma)\delta_\gamma^\lambda\Big] \\
&= \frac{1}{N_a}\Big[\sum_\alpha (E_{\alpha.} - \mathcal{E})^2 x^\alpha\Big] + \frac{1}{N_v}\Big[\sum_\gamma (E_{.\gamma} - \mathcal{E})^2 y^\gamma\Big] \\
&= \frac{1}{N_a}M_{A,2} + \frac{1}{N_v}M_{V,2}
\end{aligned} \tag{S45}$$

where $\delta_{\alpha\beta}$ is a Kronecker delta function. A similar approach finds the diffusion terms for the second moments and the cross-correlation terms between the first and the second moments in the variable and the conserved regions (see e.g., [9] for further details),

$$\mathcal{G}^{M_{A,2},M_{A,2}} = \frac{1}{N_a}(M_{A,4} - M_{A,2}^2), \qquad \mathcal{G}^{M_{V,2},M_{V,2}} = \frac{1}{N_v}(M_{V,4} - M_{V,2}^2), \qquad \mathcal{G}^{\hat{M}_{A,2},\hat{M}_{A,2}} = \frac{1}{N_a}(\hat{M}_{A,4} - \hat{M}_{A,2}^2)$$

$$\mathcal{G}^{\mathcal{E},M_{A,2}} = \frac{1}{N_a}M_{A,3}, \qquad \mathcal{G}^{\mathcal{E},M_{V,2}} = \frac{1}{N_v}M_{V,3}, \qquad \mathcal{G}^{\hat{\mathcal{E}},\hat{M}_{A,2}} = \frac{1}{N_a}\hat{M}_{A,3} \tag{S46}$$

B.3 Stochastic evolution of molecular phenotypes (linear-averaged fitness)

Putting all the evolutionary forces together, we can write down evolution equations for the statistics of binding affinities in a linear fitness landscape introduced in equations (S39, S41),

$$\textbf{variable region:} \qquad \frac{d}{dt}\mathcal{E} = -2(\mu_v + \mu_a)\mathcal{E} + S_a M_{A,2} - S_v\, M_{V,2} + \chi_{\mathcal{E}} \tag{S47}$$

$$\textbf{conserved region:} \qquad \frac{d}{dt}\hat{\mathcal{E}} = S_a\, \hat{M}_{A,2} - 2\mu_a\, \hat{\mathcal{E}} + \chi_{\hat{\mathcal{E}}} \tag{S48}$$

with the Gaussian correlated noise statistics due to the genetic drift,

$$\langle \chi_{\mathcal{E}} \rangle = 0, \qquad \langle \chi_{\mathcal{E}}(t)\chi_{\mathcal{E}}(t')\rangle = \left[\frac{M_{A,2}}{N_a} + \frac{M_{V,2}}{N_v}\right]\delta(t-t') \tag{S49}$$

$$\langle \chi_{\hat{\mathcal{E}}} \rangle = 0, \qquad \langle \chi_{\hat{\mathcal{E}}}(t)\chi_{\hat{\mathcal{E}}}(t')\rangle = \left[\frac{\hat{M}_{A,2}}{N_a}\right]\delta(t-t') \tag{S50}$$

Similarly, we can write down the stochastic evolution equations for the second moments $M_{A,2}$, $\hat{M}_{A,2}$ and $M_{V,2}$,

$$\frac{d}{dt}M_{A,2} = -4\mu_a(M_{A,2} - \ell\mathcal{K}_2) - 4\mu_v M_{A,2} - \frac{M_{A,2}}{N_a} + S_a M_{A,3} + \chi_{M_{A,2}} \tag{S51}$$

$$\frac{d}{dt}\hat{M}_{A,2} = -4\mu_a(\hat{M}_{A,2} - \hat{\ell}\hat{\mathcal{K}}_2) - \frac{\hat{M}_{A,2}}{N_a} + S_a \hat{M}_{A,3} + \chi_{\hat{M}_{A,2}} \tag{S52}$$

$$\frac{d}{dt}M_{V,2} = -4\mu_v(M_{V,2} - \ell\mathcal{K}_2) - 4\mu_a M_{V,2} - \frac{M_{V,2}}{N_v} - S_v M_{V,3} + \chi_{M_{V,2}} \tag{S53}$$

with Gaussian correlated noise statistics,

$$\langle \chi_{M_{A,2}} \rangle = 0, \qquad \langle \chi_{M_{A,2}}(t)\chi_{M_{A,2}}(t')\rangle = \left[\frac{M_{A,4} - (M_{A,2})^2}{N_a}\right]\delta(t-t') \tag{S54}$$

$$\langle \chi_{\hat{M}_{A,2}} \rangle = 0, \qquad \langle \chi_{\hat{M}_{A,2}}(t)\chi_{\hat{M}_{A,2}}(t')\rangle = \left[\frac{\hat{M}_{A,4} - (\hat{M}_{A,2})^2}{N_a}\right]\delta(t-t') \tag{S55}$$

$$\langle \chi_{M_{V,2}} \rangle = 0, \qquad \langle \chi_{M_{V,2}}(t)\chi_{M_{V,2}}(t')\rangle = \left[\frac{M_{V,4} - (M_{V,2})^2}{N_v}\right]\delta(t-t') \tag{S56}$$

$$\langle \chi_{M_{A,2}}(t)\chi_{\mathcal{E}}(t')\rangle = \frac{M_{A,3}}{N_a}\,\delta(t-t'), \qquad \langle \chi_{\hat{M}_{A,2}}(t)\chi_{\hat{\mathcal{E}}}(t')\rangle = \frac{\hat{M}_{A,3}}{N_a}\,\delta(t-t') \tag{S57}$$

$$\langle \chi_{M_{V,2}}(t)\chi_{\mathcal{E}}(t')\rangle = \frac{\langle M_{V,3}\rangle}{N_v}\,\delta(t-t') \tag{S58}$$

It should be noted that we ignore the linkage correlations between the binding affinity of the variable region $\mathcal{E}$ and conserved region $\hat{\mathcal{E}}$ of the virus. From the numerical analysis we see that the covariance between the linked variable and conserved regions, $\langle \sum_\alpha x^\alpha (\mathcal{E}_{\alpha\,.} - \mathcal{E})\,(\hat{\mathcal{E}}_\alpha - \hat{\mathcal{E}})\rangle$ is small compared to the diversity of the average binding affinity in both regions of antibody and viral populations, $\langle M_{A,2}\rangle$, $\langle \hat{M}_{A,2}\rangle$ and $\langle M_{V,2}\rangle$; S2D Fig. Lineages with access to the conserved region of the virus adapt by aligning their sites to the conserved sequence, and hence, remain relatively conserved with variations arising only from the stochastic forces of mutation and genetic drift. In Section B.4 we explicitly show that the auto-correlation time for the binding affinity in the conserved region is longer than in the variable interaction region; see equations (S82, S81). Therefore, the correlation between the binding affinity of the variable and the conserved regions remains small throughout the evolutionary process.

Stationary solutions for trait mean and diversity. From equations above we can solve for the stationary mean binding affinity, binding diversity in both populations, and the covariance between the moments as a function of the higher moments,

$$\langle\mathcal{E}\rangle = \frac{1}{2(\theta_a+\tilde{\theta}_v)} N_a S_a \langle M_{A,2}\rangle - \frac{1}{2(\tilde{\theta}_a+\theta_v)} N_v S_v \langle M_{V,2}\rangle \tag{S59}$$

$$\langle\mathcal{E},\mathcal{E}\rangle = \frac{1}{4(\theta_a+\tilde{\theta}_v)}\left[\langle M_{A,2}\rangle + 2N_a S_a\langle\mathcal{E}, M_{A,2}\rangle\right] + \frac{1}{4(\tilde{\theta}_a+\theta_v)}\left[\langle M_{V,2}\rangle - 2N_v S_v\langle\mathcal{E}, M_{V,2}\rangle\right] \tag{S60}$$

$$\langle\hat{\mathcal{E}}\rangle = N_a S_a\langle\hat{M}_{A,2}\rangle/2\theta_a \tag{S61}$$

$$\langle\hat{\mathcal{E}},\hat{\mathcal{E}}\rangle = \frac{1}{4\theta_a}\left[\langle\hat{M}_{A,2}\rangle + 2N_a S_a\langle\hat{\mathcal{E}},\hat{M}_{A,2}\rangle\right] \tag{S62}$$

$$\left\langle M_{A,2}\right\rangle = \frac{1}{1+4(\theta_a+\tilde{\theta}_v)}\Big[4\ell\mathcal{K}_2\theta_a + (N_a S_a)\langle M_{A,3}\rangle\Big] \tag{S63}$$

$$\left\langle M_{V,2}\right\rangle = \frac{1}{1+4(\tilde{\theta}_a+\theta_v)}\Big[4\ell\mathcal{K}_2\theta_v - (N_v S_v)\langle M_{V,3}\rangle\Big] \tag{S64}$$

$$\langle\mathcal{E}, M_{A,2}\rangle = \frac{1}{1+6(\theta_a+\tilde{\theta}_v)}\Big[\langle M_{A,3}\rangle + N_a S_a\left[\langle\mathcal{E}, M_{A,3}\rangle + \langle(M_{A,2})^2\rangle\right]\Big) \tag{S65}$$

$$\langle\mathcal{E}, M_{V,2}\rangle = \frac{1}{1+6(\tilde{\theta}_a+\theta_v)}\Big(\langle M_{V,3}\rangle - N_v S_v\left[\langle\mathcal{E}, M_{V,3}\rangle + \langle(M_{V,2})^2\rangle\right]\Big) \tag{S66}$$

$$\langle\mathcal{E}, M_{A,3}\rangle = \frac{\langle M_{A,4}\rangle/3 - \langle(M_{A,2})^2\rangle}{1+8/3(\theta_a+\tilde{\theta}_v)}, \qquad \langle\mathcal{E}, M_{V,3}\rangle = \frac{\langle M_{V,4}\rangle/3 - \langle(M_{V,2})^2\rangle}{1+8/3(\tilde{\theta}_a+\theta_v)} \tag{S67}$$

where $\tilde{\theta}_a = \theta_a(N_v/N_a)$ and $\tilde{\theta}_v = \theta_v(N_a/N_v)$. We denote the ensemble covariance of two stochastic variables x and y by,

$$\left\langle x, y\right\rangle \equiv \left\langle (x-\langle x\rangle)\,(y-\langle y\rangle)\right\rangle \tag{S68}$$

and hence, $\langle x, x\rangle$ is the ensemble variance of the variable x. Similar forms of the stationary solutions apply to the statistics of the binding affinity in the conserved interaction region, $\langle\hat{M}_{A,2}\rangle$, $\langle\hat{\mathcal{E}},\hat{M}_{A,2}\rangle$, and can be found by setting the viral mutation rate μ_v and the central moments $\hat{M}_{V,r}$ equal to zero in equations (S59-S67). For brevity we do not present the solutions of the central moments in the conserved region.

In equations (S47-S58), the evolution of each moment depends on the higher moments in the presence of selection, which leads to an infinite moment hierarchy. However, in the regime where rescaled coefficients satisfy $s_a\theta_a < 1$ and $s_v\theta_v < 1$, we can truncate the moment hierarchy. From the comparisons of the Wright-Fisher simulations with our theoretical results we choose to truncate the hierarchy after the 4th moment. Furthermore, higher central moments are fast stochastic variables (see e.g., [9] and the discussion in Section B.4 and S3 Fig), and their ensemble averages can sufficiently characterize the evolution of the mean binding affinity $\mathcal{E}$ and the binding diversity $M_{A,2}$, $\hat{M}_{A,2}$ and $M_{V,2}$. Therefore, we will only present ensemble-averaged equations for the 3rd and 4th moments of the phenotype distributions. In order to clarify the truncation of the moment hierarchy, we explicitly show the evolution equations and their stationary solutions for the rescaled moments of the phenotype distribution, which are defined in eq. (S22).

$$\frac{d}{d\tau_a}\langle m_{A,3}\rangle = -6\theta_a\langle m_{A,3}\rangle - 8\theta_a\left(\frac{\mathcal{K}_3}{E_0^2\mathcal{K}_1}\langle\varepsilon\rangle\right) - 6\tilde{\theta}_v\langle m_{A,3}\rangle - 3\langle m_{A,3}\rangle + s_a\left[\langle m_{A,4}\rangle - 3\left\langle(m_{A,2})^2\right\rangle\right] \tag{S69}$$

$$\frac{d}{d\tau_v}\langle m_{V,3}\rangle = -6\theta_v\langle m_{V,3}\rangle - 8\theta_v\left(\frac{\mathcal{K}_3}{E_0^2\mathcal{K}_1}\langle\varepsilon\rangle\right) - 6\tilde{\theta}_a\langle m_{V,3}\rangle - 3\langle m_{V,3}\rangle - s_v\left[\langle m_{V,4}\rangle - 3\left\langle(m_{V,2})^2\right\rangle\right] \tag{S70}$$

$$\frac{d}{d\tau_a}\langle(m_{A,2})^2\rangle = -8\theta_a\left[\langle(m_{A,2})^2\rangle - \langle m_{A,2}\rangle\right] - 8\tilde{\theta}_v\langle(m_{A,2})^2\rangle + \langle m_{A,4}\rangle - 3\left\langle(m_{A,2})^2\right\rangle \tag{S71}$$

$$\frac{d}{d\tau_v}\langle(m_{V,2})^2\rangle = -8\theta_v\left[\langle(m_{V,2})^2\rangle - \langle m_{V,2}\rangle\right] - 8\tilde{\theta}_a\langle(m_{V,2})^2\rangle + \langle m_{V,4}\rangle - 3\left\langle(m_{V,2})^2\right\rangle \tag{S72}$$

$$\frac{d}{d\tau_a}\langle m_{A,4}\rangle = -8\theta_a\left[\langle m_{A,4}\rangle - 2\frac{\mathcal{K}_4}{\ell\,\mathcal{K}_2^2} - (3-4/\ell)\langle m_{A,2}\rangle\right] - 8\tilde{\theta}_v\langle m_{A,4}\rangle + 6\left\langle(m_{A,2})^2\right\rangle - 4\langle m_{A,4}\rangle \tag{S73}$$

$$\frac{d}{d\tau_v}\langle m_{V,4}\rangle = -8\theta_v\left[\langle m_{V,4}\rangle - 2\frac{\mathcal{K}_4}{\ell\,\mathcal{K}_2^2} - (3-4/\ell)\langle m_{V,2}\rangle\right] - 8\tilde{\theta}_a\langle m_{V,4}\rangle + 6\left\langle(m_{V,2})^2\right\rangle - 4\langle m_{V,4}\rangle \tag{S74}$$

with $\tilde{\theta}_a = \theta_a(N_v/N_a)$ and $\tilde{\theta}_v = \theta_v(N_a/N_v)$. $\tau_a = t/N_a$ and $\tau_v = t/N_v$ are the evolutionary times in natural units of the neutral coalescence time in the antibody population N_a and in the viral population N_v, respectively. The term $\langle\varepsilon\rangle = 2(s_a\theta_a - s_v\theta_v\,(N_a/N_v))/(\theta_a + \theta_v\,(N_a/N_v))$ in equations (S69, S70) is the stationary solution for the rescaled mean binding affinity up to orders of $\mathcal{O}(\theta_a^2, \theta_v^2)$. The stationary solutions for the rescaled central moments of the antibody population follow,

$$\langle m_{A,2}\rangle = \frac{4\theta_a}{1+4(\theta_a+\tilde{\theta}_v)} - \frac{8\theta_a}{3+18(\theta_a+\tilde{\theta}_v)}s_a\left[\frac{\mathcal{K}_3}{E_0^2\mathcal{K}_1}\langle\varepsilon\rangle - 4s_a\theta_a^2 + \mathcal{O}(\theta_a^3)\right] + \mathcal{O}(s_a^2\theta_a^2) \tag{S75}$$

$$\langle m_{A,3}\rangle = -\frac{8}{3}\times\frac{\theta_a}{1+2(\theta_a+\tilde{\theta}_v)}\left(\frac{\mathcal{K}_3}{E_0^2\mathcal{K}_1}\langle\varepsilon\rangle\right) + \frac{32}{3}s_a\left[\theta_a^2 + \mathcal{O}(\theta_a^3)\right] + \mathcal{O}(s_a^2\theta_a^3) \tag{S76}$$

$$\left\langle(m_{A,2})^2\right\rangle = \frac{8\theta_a}{3+28\,(\theta_a+\tilde{\theta}_v)}\left[\frac{1}{\ell}\frac{\mathcal{K}_4}{\mathcal{K}_2^2} + 2\theta_a(7-4/\ell)\right] + \mathcal{O}(s_a\theta_a^3) \tag{S77}$$

$$\langle m_{A,4}\rangle = \frac{24\theta_a}{3+28\,(\theta_a+\tilde{\theta}_v)}\left[\frac{1}{\ell}\frac{\mathcal{K}_4}{\mathcal{K}_2^2} + 2\theta_a(5-4/\ell)\right] + \mathcal{O}(s_a\theta_a^3) \tag{S78}$$

Similar solutions can be found for the central moments of binding affinity in the viral population $m_{V,r}$, by replacing the subscripts a and v in the equations above. The stationary solutions for the central moments of the binding affinity in the conserved region of antibody population $\hat{m}_{A,r}$ can be found by setting the viral mutation rate equal to zero, $\theta_v = 0$, and by using the characteristics of the conserved region i.e., genetic length $\hat{\ell}$ and sites contributions $\hat{\mathcal{K}}_r$ in equations (S75-S78). S1 Fig shows a good agreement between the numerical results for the rescaled stationary mean binding affinity $\langle\varepsilon\rangle = \langle\mathcal{E}\rangle/E_0$, $\langle\hat{\varepsilon}\rangle = \langle\hat{\mathcal{E}}\rangle/\hat{E}_0$ from the Wright-Fisher simulations and the analytical solutions (S59, S61), by using the stationary ensemble averages for the diversity of the binding affinity $\langle m_{A,2}\rangle$, $\langle\hat{m}_{A,2}\rangle$ and $\langle m_{V,2}\rangle$ in eq. (S75). S2 Fig compares the analytical solution for the second central moments $\langle m_{A,2}\rangle$ and $\langle m_{V,2}\rangle$ with numerical results from the Wright-Fisher simulations, by inserting the empirical estimates of the higher moments from the simulations as in equations (S63) and (S64), (dashed lines), and by using the analytical solutions for the higher moments to estimate the stationary value for the phenotype diversity, as given by eq. (S75), (solid lines).

B.4 Time-dependent statistics and separation of time-scales

Statistics of the mean phenotype. As we show below, the higher central moments $M_{V,r}$ and $M_{A,r}$ for $(r > 1)$ are fast stochastic variables. Therefore, it is sufficient to use their stationary ensemble averages to compute the finite time correlation of the mean binding affinities, $\mathcal{E}(\tau)$ and $\hat{\mathcal{E}}(\tau)$.

The time-dependent solution for the ensemble averaged mean affinity $\langle\mathcal{E}(\tau)\rangle$ and $\langle\hat{\mathcal{E}}(\tau)\rangle$ at time τ, and the covariance between two time-points $\tau_2 \geq \tau_1$, starting from an initial condition at time $\tau_0 = 0$ with the ensemble averages for the mean affinities $\langle\mathcal{E}(0)\rangle$, $\langle\hat{\mathcal{E}}(0)\rangle$ and the diversities $\langle\mathcal{E}(0), \mathcal{E}(0)\rangle$, $\langle\hat{\mathcal{E}}(0), \hat{\mathcal{E}}(0)\rangle$ follows,

$$\langle\mathcal{E}(\tau)\rangle = (1 - \mathrm{e}^{-2(\theta_a+\tilde{\theta}_v)\tau})\,\langle\mathcal{E}\rangle + \mathrm{e}^{-2(\theta_a+\tilde{\theta}_v)\tau}\,\langle\mathcal{E}(0)\rangle \tag{S79}$$

$$\langle\hat{\mathcal{E}}(\tau)\rangle = (1 - \mathrm{e}^{-2\theta_a\tau})\,\langle\hat{\mathcal{E}}\rangle + \mathrm{e}^{-2\theta_a\tau}\,\langle\hat{\mathcal{E}}(0)\rangle \tag{S80}$$

$$\begin{aligned}\langle\mathcal{E}(\tau_1), \mathcal{E}(\tau_2)\rangle &= \mathrm{e}^{-2(\theta_a+\tilde{\theta}_v)\tau_2}\langle\mathcal{E}(0), \mathcal{E}(0)\rangle + \left[\frac{\langle M_{A,2}\rangle}{N_a} + \frac{\langle M_{V,2}\rangle}{N_v}\right]\int_0^{\tau_1} \mathrm{e}^{-2(\theta_a+\tilde{\theta}_v)(\tau_1-\tau')}\mathrm{e}^{-2(\theta_a+\tilde{\theta}_v)(\tau_2-\tau')}d\tau' \\ &= \mathrm{e}^{-2(\theta_a+\tilde{\theta}_v)\tau_2}\,\langle\mathcal{E}(0), \mathcal{E}(0)\rangle + \left[\frac{\langle M_{A,2}\rangle}{4(\theta_a+\tilde{\theta}_v)} + \frac{\langle M_{V,2}\rangle}{4(\tilde{\theta}_a+\theta_v)}\right]\left[\mathrm{e}^{-2(\theta_a+\tilde{\theta}_v)(\tau_2-\tau_1)} - \mathrm{e}^{-2(\theta_a+\tilde{\theta}_v)(\tau_1+\tau_2)}\right]\end{aligned} \tag{S81}$$

$$\langle\hat{\mathcal{E}}(\tau_1), \hat{\mathcal{E}}(\tau_2)\rangle = \mathrm{e}^{-2\theta_a\tau_2}\,\langle\hat{\mathcal{E}}(0), \hat{\mathcal{E}}(0)\rangle + \frac{\langle\hat{M}_{A,2}\rangle}{4\theta_a}\,\left[\mathrm{e}^{-2\theta_a(\tau_2-\tau_1)} - \mathrm{e}^{-2\theta_a(\tau_1+\tau_2)}\right] \tag{S82}$$

where $\langle\mathcal{E}\rangle$ and $\langle\hat{\mathcal{E}}\rangle$ are the stationary values of the mean phenotype in the variable and the conserved interaction regions, given by equations (S59, S61). Time τ is measured in units of the neutral coalescence time for antibodies, N_a. The characteristic time-scale for the decay of the mean binding affinity in the variable interaction region of the virus is $1/(2(\theta_a + \tilde{\theta}_v))$ in units of N_a, which is shorter than the time-scale for the conserved region, $1/2\theta_a$. Therefore, binding affinity in the conserved region is correlated over a longer period of time compared to the variable region (i.e., about twice as long if $\theta_a \sim \tilde{\theta}_v$). The difference in time-scale explains the small covariance due to the genetic linkage between the conserved and the variable region of the virus shown in S3 Fig.

Statistics of the phenotype diversity. As shown in [9], the fluctuations in the phenotype diversity are scale invariant, which is a consequence of coherent, genome-wide linkage-disequilibrium fluctuations in the absence of recombination. It is generated by sampling from a set of genotypes with binding affinities $E_{\alpha\,\cdot}$ in antibodies and $E_{\cdot\,\gamma}$ in viruses from the underlying distributions with variance $M_{A,2}$ and $M_{V,2}$, which scale like the genome length ℓ. These large fluctuations result in a relatively short correlation time for the phenotype diversity, shown in S3 Fig. Similar to the mean binding affinity, we can estimate the typical lifetime of these fluctuations from the stationary auto-correlation function,

$$\langle M_{A,2}(\tau_a), M_{A,2}(\tau'_a)\rangle \sim \mathrm{e}^{-(\tau_a-\tau'_a)}, \qquad \langle M_{V,2}(\tau_v), M_{V,2}(\tau'_v)\rangle \sim \mathrm{e}^{-(\tau_v-\tau'_v)} \tag{S83}$$

where τ_a, τ'_a are measured in units of the antibody neutral coalescence time N_a, and τ_v, τ'_v are measured in units of the viral neutral coalescence time N_v. S3 Fig shows the decay of the stationary auto-correlation for the diversity of the binding affinity $M_{A,2}$, $\hat{M}_{A,2}$ and $M_{V,2}$ as a function of the evolutionary separation time $\Delta\tau = \tau - \tau'$. It is evident that the characteristic decay time for the phenotype diversity (S83) is much shorter than that of the mean phenotype, given by the auto-correlation function in eqs. (S81, S82).

B.5 Alternative fitness models

Nonlinear activation probability based on average binding (nonlinear-averaged). We assume that the growth rate (fitness) of an antibody is proportional to the logarithm of its activation probability given by eq. (S38), which may be approximated by a linear function if the nonlinearity is small (S39). Here, we numerically study the effect of nonlinear sigmoidal fitness functions by comparing the evolutionary dynamics of populations in fitness

landscapes with different values of nonlinearity $\beta = \beta_0\sqrt{E_0^2 + \hat{E}_0^2}$ and binding threshold $e^* = E^*/\sqrt{E_0^2 + \hat{E}_0^2}$, while keeping the overall strength of (rescaled) selection, $s_a = c_a\beta/(1 + \exp[-\beta e^*])$ constant. The strength of selection corresponds to the slope of the approximate linear-averaged fitness function in eq. (S39).

As the rescaled nonlinearity $\beta = \beta_0 E_0$ of the fitness function (S38) increases, the mean binding affinity $\mathcal{E}$ becomes closer to the neutral value; see S4A Fig. This is due to the sigmoid form of the fitness function, which reduces fitness differences between genotypes at extreme values of binding affinity. Since mutations push the mean binding affinity towards zero, the reduced advantage of binding at the extremes moves the stationary binding affinity towards zero.

Similar arguments suggest that the rate of adaptation in the antibody population should decrease as the fitness landscapes become more non-linear. The rate of adaptation is determined by fitness flux [13, 14], and is approximately equal to the variance of fitness in the population [15]; see Section C for detailed discussion. Due to the sigmoidal shape of the fitness function, fitness differences become small at large values of binding affinity (i.e., the functional antibodies), resulting in a reduction of the fitness variance in the population, and hence, a lower rate of adaptation. However, this effect is less pronounced when the threshold for specific interaction is very large, $e^* \gg 1/\beta$. In this case, the fitness function is nearly linear for most antibodies, since their binding affinity fall below the binding threshold e^*. In this regime, the fitness variance and the rate of adaptation are only sensitive to the selection strength s_a (i.e., slope of fitness at $e = 0$), and not the nonlinearity of the fitness landscape. Evidently, the fitness variance (S4B Fig) is less sensitive to the non-linearity, than the mean binding affinity (S4A Fig).

Nonlinear activation probability based on the strongest binding (nonlinear-EVD). We study a model for activation of antibodies which is based on their *strongest binding affinity* with a subset of viruses. The basic assumption is that an antibody attempts to bind to a set of viruses (which may be smaller than the viral population size), and once a high affinity binding occurs, it begins to proliferate. Similar treatments have been introduced in the context of T-cell activation [16, 17]. The probability distribution function, $\Pi(E^*_{\alpha\,.})$ of the strongest of R independent binding interactions between the antibody $\mathbf{A}^\alpha$ and the viral population $\{\mathbf{V}\}$ can be obtained using extreme value statistics. According to extreme value theory, if the distribution of binding affinities for a given antibody has an exponential tail, the corresponding distribution for its strongest binding affinity belongs to the class of Gumbel distributions [18]. In the evolutionary regime that we study here, the amount of genetic polymorphism in the population of antibodies results in a Gaussian-like distribution for the binding affinities, with mean $E_{\alpha\,.} + \hat{E}_{\alpha\,.}$, and variance $I^{(2)}_{\alpha\,.}$ given by eq. (S11). Therefore, the corresponding probability distribution for the strongest binding affinity out of R independent trials, is a Gumbel distribution [18] with a peak at,

$$E^\alpha_{\max} = E_{\alpha\,.} + \hat{E}_{\alpha\,.} + \sqrt{2I^{(2)}_{\alpha\,.}\ln R} \tag{S84}$$

and a width $\Sigma^\alpha = \sqrt{\pi I^{(2)}_{\alpha\,.}/(12\ln R)}$. If we assume that $\ln R \gg 1$, the distribution is sharply peaked, and $E^\alpha_{\max}$ is sufficient to describe it. In addition, we assume the activation probability is a sigmoid function of $E^\alpha_{\max}$,

$$p_{A,\max}(\mathbf{A}^\alpha) = \frac{1}{1 + \exp[-\beta_0(E^\alpha_{\max} - E^*)]}. \tag{S85}$$

The fitness function $f_{A,\max}(\mathbf{A}^\alpha; \{V\})$ for the *nonlinear-EVD* model is related to the logarithm of the activation probability,

$$f_{A,\max}(\mathbf{A}^\alpha; \{V\}) \quad = \quad c_a \log[p_{\max}(\mathbf{A}^\alpha)] = -c_a \log(1 + \exp[-\beta_0(E^\alpha_{\max} - E^*)]) \tag{S86}$$

where the coefficients are similarly defined as in eq. (S38). S4A Fig shows the stationary mean binding affinity for *nonlinear-EVD* fitness model. While the mean binding affinity is sensitive to the nonlinearity parameter β, it is relatively insensitive to the number of interactions R, and behaves similarly to the nonlinear-averaged model. This is not surprising given the logarithmic dependence of binding affinity on the number of interactions R in eq. (S84).

C Fitness flux and coevolutionary transfer flux

The fitness flux $\phi(t)$ characterizes the adaptive response of a population by genotypic or phenotypic changes in a population [19, 13, 14, 20, 21]. The cumulative fitness flux, $\Phi(\tau) = \int_t^{t+\tau} N\phi(t')dt'$, measures the total amount of adaptation over an evolutionary period τ [19, 14]. The evolutionary statistics of this quantity is specified by the fitness flux theorem [14]. In our model, the fitness flux for the antibodies $\phi_A(t)$ and the viruses $\phi_V(t)$ follow,

$$\phi_A(t) = \sum_{\alpha\in\text{antibodies}} \frac{\partial F_A(t)}{\partial x^\alpha} \times \frac{dx^\alpha(t)}{dt} \tag{S87}$$

$$\phi_V(t) = \sum_{\gamma\in\text{viruses}} \frac{\partial F_V(t)}{\partial y^\gamma} \times \frac{dy^\gamma(t)}{dt} \tag{S88}$$

where, $F_A(t)$ and $F_V(t)$ are the mean fitness of the antibody and the viral populations at time t, and time is measured in units of generations. It should be noted that the cumulative fitness flux for evolution in a constant environment (equilibrium) is equal to the difference of the mean fitness between the final and the initial time points. However, the cumulative fitness flux in time-dependent environments (non-equilibrium) depends on the whole evolutionary history of the population, and captures its incremental adaptive response to the underlying environmental fluctuations.

We introduce a new measure of interaction between coevolving populations "*transfer flux*", which is the change in the mean fitness of a population due to the evolution of the opposing population. The transfer flux from antibodies to viruses $\mathcal{T}_{A\to V}$ and from viruses to antibodies $\mathcal{T}_{V\to A}$ follow,

$$\mathcal{T}_{A\to V}(t) = \sum_{\alpha\in\text{antibodies}} \frac{\partial F_V(t)}{\partial x^\alpha} \times \frac{dx^\alpha(t)}{dt} \tag{S89}$$

$$\mathcal{T}_{V\to A}(t) = \sum_{\gamma\in\text{viruses}} \frac{\partial F_A(t)}{\partial y^\gamma} \times \frac{dy^\gamma(t)}{dt} \tag{S90}$$

In the regime of substantial selection $s_a, s_v \gtrsim 1$, the transfer flux in antagonistically interacting populations of antibodies and viruses is always negative, implying that adaptation of one population reduces the fitness of the opposing population.

The fitness flux and transfer flux are rates of adaptation and interaction that are time-independent only in the stationary state. The total amount of adaptation for non-stationary evolution, where the fluxes change in time, can be generally measured by the cumulative fitness and transfer flux. For coevolution in the linear-averaged fitness landscape of equations (S39, S41) the cumulative fitness flux over an evolutionary period $[t_0 : t]$ for antibodies and viruses follow from a simple genotype-to-phenotype projection,

$$\begin{aligned}\langle\Phi_A(t_0:t)\rangle &= \left\langle N_a \int_{t'=t_0}^{t} \phi_A(t')dt' \right\rangle \\ &= \left\langle N_a \int_{t'=t_0}^{t} dt' \left(\frac{\partial F_A(t')}{\partial \mathcal{E}(t')} \frac{\partial \mathcal{E}(t')}{\partial t'}\Big|_{\{\mathbf{V}\}} + \frac{\partial F_A(t')}{\partial \hat{\mathcal{E}}(t')} \frac{\partial \hat{\mathcal{E}}(t')}{\partial t'}\Big|_{\{\mathbf{V}\}} \right) \right\rangle \\ &= \left\langle \int_{t'=t_0/N_a}^{t/N_a} dt' \left[-2\theta_a \left(s_a\varepsilon(t') + \hat{s}_a\hat{\varepsilon}(t') \right) + \left(s_a^2 m_{A,2}(t') + \hat{s}_a^2 \hat{m}_{A,2}(t') \right) \right] \right\rangle \end{aligned} \tag{S91}$$

$$\begin{aligned}\langle\Phi_V(t_0:t)\rangle &= \left\langle N_v \int_{t'=t_0 0}^{t} \phi_V(t')dt' \right\rangle \\ &= \left\langle \int_{t'=t_0/N_v}^{t/N_v} dt' \left[2\theta_v s_v \varepsilon(t') + s_v^2\, m_{V,2}(t') \right] \right\rangle \end{aligned} \tag{S92}$$

The first terms (proportional to θ) in the integrants of eqs. (S91, S92) are the fitness changes due to mutations and the second terms are due to selection; the changes due to genetic drift are zero for the ensemble-averaged fitness

flux of the linear-averaged fitness landscapes in eqs. (S39, S41). In the regime of substantial selection $s_a, s_v \gtrsim 1$, the fitness flux in a polymorphic population asymptotically converges to the variance of the stationary fitness distribution in the population (e.g., $s_a^2\, m_{A,2} + \hat{s}_a^2\, \hat{m}_{A,2}$ for antibodies) [14], which is in accordance with the rate of adaptation given by Fisher's fundamental theorem and Price's equation [15, 22].

Similarly, the cumulative transfer fluxes over an evolutionary period $[t_0 : t]$ read,

$$\begin{aligned}\langle \mathbf{T}_{A\to V}(t_0:t)\rangle &= \left\langle N_v \int_{t'=t_0}^{t} \mathcal{T}_{A\to V}(t')dt' \right\rangle \\ &= \left\langle N_v \int_{t'=t_0}^{t} \left(\frac{\partial F_V(t')}{\partial \mathcal{E}(t')} \frac{\partial \mathcal{E}(t')}{\partial t'}\Big|_{\{\mathbf{V}\}} + \frac{\partial F_V(t')}{\partial \hat{\mathcal{E}}(t')} \frac{\partial \hat{\mathcal{E}}(t')}{\partial t'}\Big|_{\{\mathbf{V}\}} \right) \right\rangle \\ &= (N_v/N_a) \left\langle \int_{t'=t_0/N_v}^{t/N_v} dt' \, \left[2\theta_a s_v \big(\varepsilon(t') + \hat{\varepsilon}(t')\big) - s_v \left(s_a m_{A,2}(t') + \hat{s}_a \hat{m}_{A,2}(t') \right) \right] \right\rangle \end{aligned} \tag{S93}$$

$$\begin{aligned}\langle \mathbf{T}_{V\to A}(t_0:t)\rangle &= \left\langle N_a \int_{t'=t_0}^{t} \mathcal{T}_{V\to A}(t')dt' \right\rangle \\ &= (N_a/N_v) \left\langle \int_{t'=t_0/N_a}^{t/N_a} dt' \, \left[-2\theta_v s_a \varepsilon(t') - s_a s_v \, m_{V,2}(t') \right] \right\rangle \end{aligned} \tag{S94}$$

The first terms in equations (S93, S94) are the fitness changes due to mutation, the second terms are due to selection.

In the stationary state, the cumulative flux values grow linearly with the evolutionary time, and simplify to,

$$\langle \Phi_A(\tau_a)\rangle_{\text{st.}} = -\langle \mathbf{T}_{V\to A}(\tau_a)\rangle_{\text{st.}} = \frac{s_a}{\tilde{\theta}_a + \theta_v} \left(s_a \langle m_{A,2}\rangle \theta_v + s_v \langle m_{V,2}\rangle \theta_a \right) \tau_a \tag{S95}$$

$$\langle \Phi_V(\tau_v)\rangle_{\text{st.}} = -\langle \mathbf{T}_{A\to V}(\tau_v)\rangle_{\text{st.}} = \frac{s_v}{\tilde{\theta}_v + \theta_a} \left(s_a \langle m_{A,2}\rangle \theta_v + s_v \langle m_{V,2}\rangle \theta_a \right) \tau_v \tag{S96}$$

where we have substituted the expected values for the ensemble averaged binding affinities in the stationary state, given by eqs. (S59, S61). $\tau_a = (t - t_0)/N_a$ and $\tau_v = (t - t_0)/N_v$ are the evolutionary times respectively in natural units of the neutral coalescence time in the antibody population N_a and in the viral population N_v. In the stationary state, the fitness flux in each population and the transfer flux from the opposing population sum up to 0, keeping the mean fitness of both populations constant. Non-stationary states occur during transient evolutionary dynamics of the whole population, or when considering a subset of the population, such as a clonal lineage, whose size fluctuates to fixation or extinction. In particular, the imbalance between the fitness flux and the transfer flux may determine the evolutionary fate of a clonal lineage, which we discuss in Section E. A convenient way to measure fitness and transfer flux is from time-shifted fitness measurements, for the stationary (Fig 4 and S5 Fig) and non-stationary (S6 Fig) conditions.

D Signature of coevolution from time-shifted fitness measurements

Measuring interactions between antibody and viral populations sampled at different time points provides means to quantify the amount of host-pathogen co-adaptation. We introduce the time-shifted binding affinity between viruses at time t and antibodies at time $t + \tau$ in the variable and in the conserved regions,

$$\mathcal{E}_\tau(t) = \sum_{\alpha,\gamma} E_{\alpha\gamma} x^\alpha(t+\tau)\, y^\gamma(t) \tag{S97}$$

$$\hat{\mathcal{E}}_\tau(t) = \sum_{\alpha,\gamma} \hat{E}_\alpha x^\alpha(t+\tau) y^\gamma(t) = \hat{\mathcal{E}}(t+\tau) \tag{S98}$$

and the corresponding rescaled quantities, $\varepsilon_\tau(t) = \mathcal{E}_\tau(t)/E_0$ and $\hat{\varepsilon}_\tau(t) = \hat{\mathcal{E}}_\tau(t)/\hat{E}_0$. Since the virus cannot evolve in the conserved region, the time-shifted binding affinity in this region $\hat{\mathcal{E}}_\tau(t)$ is identical to the non-shifted affinity $\hat{\mathcal{E}}(t+\tau)$ at time $t+\tau$. The time-shifted fitness for antibodies and viruses at time t in interaction with the opposing population sampled at time $t+\tau$ follow,

- time-shifted viral fitness:

$$N_v F_{V;\tau}(t) \quad = \quad -s_v \varepsilon_\tau(t) \tag{S99}$$

- time-shifted antibody fitness:

$$N_a F_{A;\tau}(t) \quad = \quad s_a \varepsilon_{-\tau}(t+\tau) + \hat{s}_a \hat{\varepsilon}(t) \tag{S100}$$

As shown in Fig 4 in the main text, the behavior of the time-shifted binding affinity (or fitness) is primarily determined by the strength of selection on the phenotype at short values of time-lag τ, and is characterized by randomizing mutations at large separation times. Here, we analytically characterize the stationary state behavior of the time-shifted binding affinity as a function of the separation time τ. The change in time-shifted binding affinity due to the affinity maturation of antibodies (adaptation) to neutralize the focal viral population (i.e., for positive separation times $\tau > 0$) follows,

$$\begin{aligned}
\frac{d}{d\tau}\langle \varepsilon_\tau(t)\rangle &= \left\langle \frac{1}{E_0}\sum_{\alpha,\gamma} E_{\alpha\,\gamma} y^\gamma(t)\,\frac{d}{d\tau} x^\alpha(t+\tau)\right\rangle \\
&= -2\tilde{\theta}_a \langle \varepsilon_\tau(t)\rangle + \left\langle \frac{s_a}{E_0^2}\sum_{\alpha} E_{\alpha\,\cdot}(t)\,(E_{\alpha\,\cdot}(t+\tau) - \mathcal{E}(t+\tau))\, x^\alpha(t+\tau)\right\rangle \\
&\simeq -2\tilde{\theta}_a \langle \varepsilon_\tau(t)\rangle + s_a \langle m_{A,2}\rangle e^{-2\theta_v \tau}
\end{aligned} \tag{S101}$$

where time is measured in units of the viral coalescence time, N_v. We used a mean-field approach in the stationary state to approximate the finite-time divergence of the averaged binding affinity for a given antibody in a time-varying environment of evolving viruses, i.e., $\langle \sum_\alpha x^\alpha(t+\tau)(E_{\alpha\,\cdot}(t+\tau) - \mathcal{E}(t+\tau))\,(E_{\alpha\,\cdot}(t) - \mathcal{E}(t))\rangle \simeq e^{-2\theta_v \tau}\,\langle \sum_\alpha x^\alpha(t+\tau)(E_{\alpha\,\cdot}(t+\tau) -$ $e^{-2\theta_v \tau}\langle M_{A,2}\rangle$. The behavior of the time-shifted binding affinity at negative separation times $\tau < 0$ is mainly determined by the adaptation (escape) of the viruses to the antibodies in the past. In the stationary state, the backward dynamics of the time-shifted binding affinity with respect to the focal viral population is equivalent to the forward dynamics with respect to the focal antibody population, which can be evaluated similarly to eq. (S101). Combining the forward and the backward dynamics results in the following functional form for the rescaled time-shifted binding affinity,

- for antibody affinity maturation, $\tau \geq 0$

$$\langle \varepsilon_\tau(t)\rangle = \begin{cases} \frac{s_a m_{A,2}}{2(\theta_a - \tilde{\theta}_v)}\, e^{-2\theta_v \tau} - \left(\frac{s_v m_{V,2}}{2(\tilde{\theta}_a + \theta_v)} + \frac{\theta_v s_a m_{A,2}}{\theta_a \tilde{\theta}_a - \theta_v \tilde{\theta}_v}\right) e^{-2\tilde{\theta}_a \tau} & \theta_a \neq \theta_v \\ \\ \left(\frac{s_a m_{A,2}(N_v/N_a) - s_v m_{V,2}}{4\theta} + s_a m_{A,2}(N_v/N_a)\tau\right) e^{-2\theta\tau} & \tilde{\theta}_a = \theta_v = \theta \end{cases} \tag{S102}$$

- for viral escape, $\tau < 0$

$$\langle \varepsilon_\tau(t)\rangle = \begin{cases} \frac{s_v m_{V,2}}{2(\tilde{\theta}_a - \theta_v)}\, e^{-2\tilde{\theta}_a |\tau|} + \left(\frac{s_a m_{A,2}}{2(\theta_a + \tilde{\theta}_v)} - \frac{\theta_a s_v m_{V,2}}{\theta_a \tilde{\theta}_a - \theta_v \tilde{\theta}_v}\right) e^{-2\theta_v |\tau|} & \theta_a \neq \theta_v \\ \\ \left(\frac{s_a m_{A,2}(N_v/N_a) - s_v m_{V,2}}{4\theta} - s_v m_{V,2}|\tau|\right) e^{-2\theta|\tau|} & \tilde{\theta}_a = \theta_v = \theta \end{cases} \tag{S103}$$

with $\tilde{\theta}_a = \theta_a(N_v/N_a)$ and $\tilde{\theta}_v = \theta_v(N_a/N_v)$. S5 Fig shows a good agreement between the numerical results for the time-shifted fitness $N_v\langle F_{V;\tau}\rangle = -s_v\langle \varepsilon_\tau\rangle$ from the Wright-Fisher simulations and the analytical solutions (S102, S103), in the stationary state. The slope of time-shifted fitness at time-lag $\tau = 0$ is a measure of the antibody population's fitness flux (towards the past) and the transfer flux from the opposing population (towards the future), which are equal in stationary state as depicted in S5 Fig. In the non-stationary state, the time-shifted fitness $\langle F_{V;\tau}(t)\rangle$ may have a discontinuous derivative at $\tau = 0$, due to an imbalance between fitness flux and transfer flux (S6 Fig).

E Evolution of multiple antibody lineages

Fixation probability in a general fitness landscape. We extend our results to multiple clonal antibody lineages evolving with a viral population. We denote the frequency of an antibody lineage with size $N_a^{\mathcal{C}}$ by $\rho^{\mathcal{C}} = N_a^{\mathcal{C}}/N_a$. Assuming that mutations cannot change the identity of one lineage to another, the growth of a given lineage $\mathcal{C}$ depends on the relative mean fitness of the lineage $F_{A^{\mathcal{C}}}$ to the mean fitness of the whole population $F_A(t) = \sum_{\mathcal{C}} F_{A^{\mathcal{C}}}(t)\rho_{\mathcal{C}}(t)$, and on the strength of stochasticity due to genetic drift,

$$\frac{d}{dt}\rho_{\mathcal{C}}(t) = \sum_{\alpha} \left(f_{\mathcal{C}^\alpha}(t) - F_A(t)\right) x^\alpha_{\mathcal{C}}(t) + \sqrt{\frac{\rho_{\mathcal{C}}(1-\rho_{\mathcal{C}})}{N_a}} \tag{S104}$$

where $f_{\mathcal{C}^\alpha}(t)$ is the fitness of the genotype $\mathbf{A}^\alpha$ in the lineage $\mathcal{C}$, and $x^\alpha_{\mathcal{C}} \equiv x_{\mathcal{C}}(\mathbf{A}^\alpha)$ is the frequency of the genotype $\mathbf{A}^\alpha$ from lineage $\mathcal{C}$ in the total population. Similar to the evolution of a single lineage, the growth of multiple lineages follows an infinite hierarchy of moment equations for the fitness distribution. Here, we truncate these equations at the second central moment of fitness, which relates to the lineage-specific fitness flux $\phi_{A^{\mathcal{C}}}$ and the transfer flux $\mathcal{T}_{V\to A^{\mathcal{C}}}$. The changes of the ensemble-averaged mean fitness of a lineage $F_{A^{\mathcal{C}}}(t)$ and the mean fitness of the whole population $F_A(t)$, weighted by the lineage frequency $\rho_{\mathcal{C}}(t)$ follow,

$$\begin{aligned}\left\langle \frac{d}{dt}\sum_{\alpha} f_{\mathcal{C}^\alpha}(t)\, x^\alpha_{\mathcal{C}}(t)\right\rangle &= \left\langle \rho_{\mathcal{C}}(t)\sum_{\alpha\in\mathcal{C}} \frac{\partial F_{A^{\mathcal{C}}}}{\partial x^\alpha_{\mathcal{C}}} \times \frac{dx^\alpha_{\mathcal{C}}}{dt}\right\rangle + \left\langle \rho_{\mathcal{C}}(t)\sum_{\gamma} \frac{\partial F_{A^{\mathcal{C}}}}{\partial y^\gamma} \times \frac{dy^\gamma}{dt}\right\rangle - \frac{1}{N_a}\left\langle F_{A^{\mathcal{C}}}(t)\,\rho_{\mathcal{C}}(t)\right\rangle \\ &\equiv \left\langle \rho_{\mathcal{C}}(t)\,\phi_{A^{\mathcal{C}}}(t)\right\rangle + \left\langle \rho_{\mathcal{C}}(t)\,\mathcal{T}_{V\to A^{\mathcal{C}}}(t)\right\rangle - \frac{1}{N_a}\left\langle F_{A^{\mathcal{C}}}(t)\,\rho_{\mathcal{C}}(t)\right\rangle \end{aligned} \tag{S105}$$

$$\left\langle \frac{d}{dt}\sum_{\alpha} F_A(t)\, x^\alpha_{\mathcal{C}}(t)\right\rangle = \left\langle \rho_{\mathcal{C}}(t)\,\phi_A(t)\right\rangle + \left\langle \rho_{\mathcal{C}}(t)\,\mathcal{T}_{V\to A}(t)\right\rangle - \frac{1}{N_a}\left\langle F_A(t)\,\rho_{\mathcal{C}}(t)\right\rangle \tag{S106}$$

Here, we assume that the mean fitness of a lineage only depends on the genotypes within the lineage, as is the case for the fitness functions given by eqs. (S39, S41). The ensemble-averaged changes of the fitness flux and the transfer flux due to selection depend on higher central moments of the fitness distribution, which we neglect in our analysis. The effects of mutation and genetic drift (using Itô calculus) on the flux quantities follow,

$$\frac{d}{dt}\left\langle \rho_{\mathcal{C}}(t)\,\phi_{A^{\mathcal{C}}}(t)\right\rangle \simeq \left\langle \rho_{\mathcal{C}}(t)\, m_{A^\alpha}\frac{\partial}{\partial x^\alpha}\phi_{A^{\mathcal{C}}}(t)\right\rangle + \frac{1}{N_a}\left[\left\langle \rho_{\mathcal{C}}(t)\,\phi_A(t)\right\rangle - 2\left\langle \rho_{\mathcal{C}}(t)\,\phi_{A^{\mathcal{C}}}(t)\right\rangle\right] \tag{S107}$$

$$\frac{d}{dt}\left\langle \rho_{\mathcal{C}}(t)\,\phi_A(t)\right\rangle \simeq \left\langle \rho_{\mathcal{C}}(t)\, m_{A^\alpha}\frac{\partial}{\partial x^\alpha}\phi_A(t)\right\rangle + \frac{1}{N_a}\left[\left\langle \rho_{\mathcal{C}}(t)\,\phi_{A^{\mathcal{C}}}(t)\right\rangle - 2\left\langle \rho_{\mathcal{C}}(t)\,\phi_A(t)\right\rangle\right] \tag{S108}$$

$$\frac{d}{dt}\left\langle \rho_{\mathcal{C}}(t)\,\mathcal{T}_{V\to A^{\mathcal{C}}}(t)\right\rangle \simeq \left\langle \rho_{\mathcal{C}}(t)\left[m_{A^\alpha}\frac{\partial}{\partial x^\alpha}\mathcal{T}_{V\to A^{\mathcal{C}}}(t) + m_{V^\gamma}\frac{\partial}{\partial y^\gamma}\mathcal{T}_{V\to A^{\mathcal{C}}}(t)\right]\right\rangle - \frac{1}{N_v}\left\langle \rho_{\mathcal{C}}(t)\,\mathcal{T}_{V\to A^{\mathcal{C}}}(t)\right\rangle \tag{S109}$$

$$\frac{d}{dt}\left\langle \rho_{\mathcal{C}}(t)\,\mathcal{T}_{V\to A}(t)\right\rangle \simeq \left\langle \rho_{\mathcal{C}}(t)\left[m_{A^\alpha}\frac{\partial}{\partial x^\alpha}\mathcal{T}_{V\to}(t) + m_{V^\gamma}\frac{\partial}{\partial y^\gamma}\mathcal{T}_{V\to A}(t)\right]\right\rangle - \frac{1}{N_v}\left\langle \rho_{\mathcal{C}}(t)\,\mathcal{T}_{V\to A}(t)\right\rangle \tag{S110}$$

where m_{A^α} and m_{V^γ} are the mutational fields associated with the changes in genotype frequencies due to mutations in antibodies and viruses, as defined by eq. (S1).

In order to compute the fixation probability $P_{\text{fix}} = \lim_{t\to\infty}\langle\rho_{\mathcal{C}}(t)\rangle$, it is convenient to use the Laplace transform of the lineage frequency, and compute its asymptotic behavior at large time (see e.g., [23]). The Laplace transform of a given function $A(t)$ can be computed as, $\mathcal{A}(z) = \sum_t A(t)\exp[-zt]$ with the inverse transform: $A(t) = \lim\limits_{T\to\infty}\frac{1}{2\pi i}\int_{\gamma-iT}^{\gamma+iT}\exp[zt]\mathcal{A}(z)$. Following this procedure for the hierarchy of equations (S104-S110) entails a general form for the fixation probability of a lineage, depending on the initial states of the antibody and the viral populations,

$$P_{\rm fix}(\mathcal{C}) = \lim_{t\to\infty} \langle \rho_c(t)\rangle$$
$$= \left\langle \rho_c(0)\right\rangle + \left\langle N_a\big(F_{A^{\mathcal{C}}}(0) - F(0)\big)\,\rho_c(0)\right\rangle + \frac{1}{3}\left\langle N_a^2\big(\phi_{A^{\mathcal{C}}}(0) - \phi_A(0)\big)\,\rho_c(0)\right\rangle$$
$$- \left\langle N_a N_v \Big(\big|\mathcal{T}_{V\to A^{\mathcal{C}}}(0)\big| - \big|\mathcal{T}_{V\to A}(0)\big|\Big)\,\rho_c(0)\right\rangle + \mathcal{O}(\theta\langle(N\delta f)^2\rangle, \langle(N\delta f)^3\rangle) \tag{S111}$$

where $\langle(\delta f)^r\rangle$ denotes the rth central moment of the fitness distribution. Here, we have neglected the change in fitness and transfer flux due to mutations, which is of the order of $\mathcal{O}(\theta\langle(N\delta f)^2\rangle)$. Below, we will explicitly study the mutational terms for the specific case of the linear fitness model in eqs. (S39, S41). The first term in eq. (S111) is the ensemble-averaged initial frequency of the lineage at time $t = 0$, and equals its fixation probability in neutrality. In the presence of selection, lineages of antibodies with higher relative mean fitness, $F_{A^{\mathcal{C}}}(0) - F(0)$, higher rate of adaptation, $\phi_{A^{\mathcal{C}}}(0) - \phi_A(0)$, and lower (absolute) transfer flux from viruses, $\big|\mathcal{T}_{V\to A^{\mathcal{C}}}(0)\big| - \big|\mathcal{T}_{V\to A}(0)\big|$, tend to dominate the population.

Fixation probability in the linear fitness landscape. In the linear-averaged fitness model (S39, S41), the growth of a lineage depends on its relative binding affinity compared to the rest of the population. In order to quantify the competition between the lineages, we define the following lineage-specific moments,

$$\overset{c}{L}_{A_m} = \langle \sum_\alpha (E_{\alpha\,\cdot} - \mathcal{E})^m\, x^\alpha_{c}\rangle, \qquad \hat{\overset{c}{L}}_{A_m} = \langle \sum_\alpha (\hat{E}_{\alpha\,\cdot} - \hat{\mathcal{E}})^m\, x^\alpha_{c}\rangle \tag{S112}$$
$$\overset{c}{L}_{A_{(m;n)}} = \left\langle \sum_\alpha (E_{\alpha\,\cdot} - \mathcal{E})^m\, x^\alpha_{c} \sum_{\beta,\mathcal{C}'} (E_{\beta\,\cdot} - \mathcal{E})^n\, x^\beta_{c'}\right\rangle \tag{S113}$$
$$\hat{\overset{c}{L}}_{A_{(m;n)}} = \left\langle \sum_\alpha (\hat{E}_{\alpha\,\cdot} - \hat{\mathcal{E}})^m\, x^\alpha_{c} \sum_{\beta,\mathcal{C}'} (\hat{E}_{\beta\,\cdot} - \hat{\mathcal{E}})^n\, x^\beta_{c'}\right\rangle \tag{S114}$$
$$\overset{c}{L}_{A_m,V_k} = \left\langle \sum_\gamma (E_{\cdot\,\gamma} - \mathcal{E})^k y^\gamma \sum_\alpha (E_{\alpha\,\gamma} - \mathcal{E})^m\, x^\alpha_{c}\right\rangle \tag{S115}$$

In this notation the zeroth order lineage-specific moment is equal to the ensemble-averaged frequency of the focal lineage $\overset{c}{L}_{A_0} \equiv \langle \rho_c\rangle$. As given by eq. (S107), the change in the frequency of the lineage $\mathcal{C}$ follows from the evolution equation,

$$\frac{d}{dt}\overset{c}{L}_{A_0} = S_a(\overset{c}{L}_{A_1} + \hat{\overset{c}{L}}_{A_1}) \tag{S116}$$

The evolutionary dynamics of multiple lineages follows from an infinite hierarchy of moment equations. In order to estimate the fixation probability of a lineage up to the order of $\mathcal{O}((NS)^2)$, it is sufficient to truncate the hierarchy at the second moment. These hierarchy of evolution equations for the lineage-specific moments $\overset{c}{L}_{A_m}$ and the cross-statistics $\overset{c}{L}_{A_m,V_k}$ follow,

variable region:

$$\frac{d}{dt}\overset{c}{L}_{A_1} = S_a\left(\overset{c}{L}_{A_2} - \overset{c}{L}_{A_{(0;2)}}\right) - S_v\left(\overset{c}{L}_{A_1,V_1} - \overset{c}{L}_{A_0,V_2}\right) - 2(\mu_a+\mu_v)\overset{c}{L}_{A_1} - \frac{\overset{c}{L}_{A_1}}{N_a} \tag{S117}$$

$$\frac{d}{dt}\overset{c}{L}_{A_2} = -4\mu_a\left(\overset{c}{L}_{A_2} - \ell\, Q_2^{\mathcal{C}}\right) - 4\mu_v\overset{c}{L}_{A_2} + \frac{\overset{c}{L}_{A_{(0;2)}} - 2\overset{c}{L}_{A_2}}{N_a} + \mathcal{O}(S_a) \tag{S118}$$

$$\frac{d}{dt}\overset{c}{L}_{A_{(0;2)}} = -4\mu_a\left(\overset{c}{L}_{A_{(0;2)}} - \ell\, Q_{(0;2)}^{\mathcal{C}}\right) - 4\mu_v\overset{c}{L}_{A_{(0;2)}} + \frac{\overset{c}{L}_{A_2} - 2\overset{c}{L}_{A_{(0;2)}}}{N_a} + \mathcal{O}(S_a) \tag{S119}$$

$$\frac{d}{dt}\overset{c}{L}_{A_1,V_1} = -4\mu_a\overset{c}{L}_{A_1,V_1} - 4\mu_v\left(\overset{c}{L}_{A_1,V_1} - \ell\sqrt{Q_2^{\mathcal{C}}\, Q_{(0;2)}^{\mathcal{C}}}\right) - \frac{\overset{c}{L}_{A_1,V_1}}{N_v} + \mathcal{O}(S_a,S_v) \tag{S120}$$

$$\frac{d}{dt}\overset{c}{L}_{A_0,V_2} = -4\mu_a\overset{c}{L}_{A_0,V_2} - 4\mu_v\left(\overset{c}{L}_{A_0,V_2} - \ell\, Q_{(0;2)}^{\mathcal{C}}\right) - \frac{\overset{c}{L}_{A_0,V_2}}{N_v} + \mathcal{O}(S_a,S_v) \tag{S121}$$

conserved region:

$$\frac{d}{dt}\hat{\overset{c}{L}}_{A_1} = S_a\left(\hat{\overset{c}{L}}_{A_2} - \hat{\overset{c}{L}}_{A_{(0;2)}}\right) - 2\mu_a\hat{\overset{c}{L}}_{A_1} - \frac{\hat{\overset{c}{L}}_{A_1}}{N_a} \tag{S122}$$

$$\frac{d}{dt}\hat{\overset{c}{L}}_{A_2} = -4\mu_a\left(\hat{\overset{c}{L}}_{A_2} - \hat{\ell}\,\hat{Q}_2^{\mathcal{C}}\right) + \frac{\hat{\overset{c}{L}}_{A_{(0;2)}} - 2\hat{\overset{c}{L}}_{A_2}}{N_a} + \mathcal{O}(S_a) \tag{S123}$$

$$\frac{d}{dt}\hat{\overset{c}{L}}_{A_{(0;2)}} = -4\mu_a\left(\hat{\overset{c}{L}}_{A_{(0;2)}} - \hat{\ell}\,\hat{Q}_{(0;2)}^{\mathcal{C}}\right) + \frac{\hat{\overset{c}{L}}_{A_2} - 2\hat{\overset{c}{L}}_{A_{(0;2)}}}{N_a} + \mathcal{O}(S_a) \tag{S124}$$

with the lineage-specific statistics of the trait scale,

$$Q_2^{\mathcal{C}} = \left\langle \rho_c\,\mathcal{K}_2^{\mathcal{C}}\right\rangle, \qquad Q_{(0;2)}^{\mathcal{C}} = \left\langle \rho_c \sum_{\text{lineages } \mathcal{C}'} \mathcal{K}_2^{\mathcal{C}'}\rho_{c'}\right\rangle \tag{S125}$$

$$\hat{Q}_2^{\mathcal{C}} = \left\langle \rho_c\,\hat{\mathcal{K}}_2^{\mathcal{C}}\right\rangle, \qquad \hat{Q}_{(0;2)}^{\mathcal{C}} = \left\langle \rho_c \sum_{\text{lineages } \mathcal{C}'} \hat{\mathcal{K}}_2^{\mathcal{C}'}\rho_{c'}\right\rangle \tag{S126}$$

$\mathcal{K}_2^{\mathcal{C}} = \sum_{i=1}^{\ell}(\kappa_i^{\mathcal{C}})^2/\ell$ and $\hat{\mathcal{K}}_2^{\mathcal{C}} = \sum_{i=1+\ell}^{\ell+\hat{\ell}}(\hat{\kappa}_i^{\mathcal{C}})^2/\hat{\ell}$ are the averaged accessibilities for a given lineage $\mathcal{C}$, similar to the definition in eq. (S21). As indicated by eqs. (S117-S124), the composite lineage-specific trait statistics $Q_2^{\mathcal{C}} - Q_{(0;2)}^{\mathcal{C}}$ and $\hat{Q}_2^{\mathcal{C}} - \hat{Q}_{(0;2)}^{\mathcal{C}}$ influence the evolution of the lineage frequency. These quantities vary over time due to changes in the lineage composition of the population,

$$\frac{d}{dt}\left(Q_2^{\mathcal{C}} - Q_{(0;2)}^{\mathcal{C}}\right) = \frac{1}{N_a}\left(Q_2^{\mathcal{C}} - Q_{(0;2)}^{\mathcal{C}}\right) + \mathcal{O}(S_a) \tag{S127}$$

$$\frac{d}{dt}\left(\hat{Q}_2^{\mathcal{C}} - \hat{Q}_{(0;2)}^{\mathcal{C}}\right) = \frac{1}{N_a}\left(\hat{Q}_2^{\mathcal{C}} - \hat{Q}_{(0;2)}^{\mathcal{C}}\right) + \mathcal{O}(S_a) \tag{S128}$$

In order to compute the fixation probability, we use the Laplace transform of the lineage-specific moments $\mathcal{L}_{A_m,V_k}^{\mathcal{C}}(z)$ and the lineage-specific statistics of the trait scale $\mathcal{Q}_2^{\mathcal{C}}(z) - \mathcal{Q}_{(0;2)}^{\mathcal{C}}(z)$, and compute the asymptotic behavior of the 0th moment $\overset{c}{L}_0$, after the inverse transform (see e.g., [23, 12]). The Laplace transform of the moment hierarchy (S117-S124) up to order of $\mathcal{O}((NS)^2)$ in $\overset{c}{\mathcal{L}}_{A_0}$ follows,

$$z\mathcal{L}^{c}_{A_0}(z) - L^{c}_{A,0}(0) = S_a(\mathcal{L}^{c}_{A_1}(z) + \hat{\mathcal{L}}^{c}_{1}(z)) \tag{S129}$$

variable region:

$$z\mathcal{L}^{c}_{A_1}(z) - L^{c}_{A_1}(0) = S_a(\mathcal{L}^{c}_{A_2}(z) - \mathcal{L}^{c}_{A_{(0;2)}}(z)) - S_v(\mathcal{L}^{c}_{A_1,V_1} - \mathcal{L}^{c}_{A_0,V_2}) - 2(\mu_a + \mu_v)\mathcal{L}^{c}_{A_1}(z) - \frac{\mathcal{L}^{c}_{A_1}(z)}{N_a} \tag{S130}$$

$$z\mathcal{L}^{c}_{A_2}(z) - L^{c}_{A_2}(0) = -4\mu_a\left(\mathcal{L}^{c}_{A_2}(z) - \ell\,\mathcal{Q}^{\mathcal{C}}_{2}(z)\right) - 4\mu_v\mathcal{L}^{c}_{A_2}(z) + \frac{\mathcal{L}^{c}_{A_{(0;2)}}(z) - 2\mathcal{L}^{c}_{A_2}(z)}{N_a} \tag{S131}$$

$$z\mathcal{L}^{c}_{A_{(0;2)}} - L^{c}_{A_{(0;2)}}(0) = -4\mu_a\left(\mathcal{L}^{c}_{A_{(0;2)}} - \ell\,\mathcal{Q}^{\mathcal{C}}_{(0;2)}(z)\right) - 4\mu_v\mathcal{L}^{c}_{A_{(0;2)}} + \frac{\mathcal{L}^{c}_{A_2} - 2\mathcal{L}^{c}_{A_{(0;2)}}}{N_a} \tag{S132}$$

$$z\mathcal{L}^{c}_{A_1,V_1} - L^{c}_{A_1,V_1}(0) = -4\mu_a\mathcal{L}^{c}_{A_1,V_1} - 4\mu_v\left(\mathcal{L}^{c}_{A_1,V_1} - \ell\sqrt{\mathcal{Q}^{\mathcal{C}}_{2}(z)\mathcal{Q}^{\mathcal{C}}_{(0;2)}(z)}\;\right) - \frac{\mathcal{L}^{c}_{A_1,V_1}}{N_v} \tag{S133}$$

$$z\mathcal{L}^{c}_{A_0,V_2} - L^{c}_{A_0,V_2}(0) = -4\mu_a\mathcal{L}^{c}_{A_0,V_2} - 4\mu_v\left(\mathcal{L}^{c}_{A_0,V_2} - \ell\mathcal{Q}^{\mathcal{C}}_{(0;2)}(z)\right) - \frac{\mathcal{L}^{c}_{A_0,V_2}}{N_v} \tag{S134}$$

$$z\left(\mathcal{Q}^{\mathcal{C}}_{2}(z) - \mathcal{Q}^{\mathcal{C}}_{(0;2)}(z)\right) - \left(Q^{\mathcal{C}}_{2}(0) - Q^{\mathcal{C}}_{(0;2)}(0)\right) = \frac{1}{N_a}\left(\mathcal{Q}^{\mathcal{C}}_{2}(z) - \mathcal{Q}^{\mathcal{C}}_{(0;2)}(z)\right) \tag{S135}$$

conserved region:

$$z\hat{\mathcal{L}}^{c}_{A_1}(z) - \hat{L}^{c}_{A_1}(0) = S_a(\hat{\mathcal{L}}^{c}_{A_2}(z) - \hat{\mathcal{L}}^{c}_{A_{(0;2)}}(z)) - 2\mu_a\hat{\mathcal{L}}^{c}_{A_1}(z) - \frac{\hat{\mathcal{L}}^{c}_{A_1}(z)}{N_a} \tag{S136}$$

$$z\hat{\mathcal{L}}^{c}_{A_2}(z) - \hat{L}^{c}_{A_2}(0) = -4\mu_a\left(\hat{\mathcal{L}}^{c}_{A_2}(z) - \hat{\ell}\,\hat{\mathcal{Q}}^{\mathcal{C}}_{2}(z)\right) + \frac{\hat{\mathcal{L}}^{c}_{A_{(0;2)}}(z) - 2\hat{\mathcal{L}}^{c}_{A_2}(z)}{N_a} \tag{S137}$$

$$z\hat{\mathcal{L}}^{c}_{A_{(0;2)}} - \hat{L}^{c}_{A_{(0;2)}}(0) = -4\mu_a\left(\hat{\mathcal{L}}^{c}_{A_{(0;2)}} - \hat{\ell}\,\hat{\mathcal{Q}}^{\mathcal{C}}_{(0;2)}(z)\right) + \frac{\hat{\mathcal{L}}^{c}_{A_2} - 2\hat{\mathcal{L}}^{c}_{A_{(0;2)}}}{N_a} \tag{S138}$$

$$z\left(\hat{\mathcal{Q}}^{\mathcal{C}}_{2}(z) - \hat{\mathcal{Q}}^{\mathcal{C}}_{(0;2)}(z)\right) - \left(\hat{Q}^{\mathcal{C}}_{2}(0) - \hat{Q}^{\mathcal{C}}_{(0;2)}(0)\right) = \frac{1}{N_a}\left(\hat{\mathcal{Q}}^{\mathcal{C}}_{2}(z) - \hat{\mathcal{Q}}^{\mathcal{C}}_{(0;2)}(z)\right) \tag{S139}$$

The inverse transform of $\mathcal{L}^{c}_{A_0}(z)$ in the limit of $z \to 0$ results in the asymptotic behavior of the ensemble-averaged frequency of the lineage $\mathcal{C}$, $\lim_{t\to\infty} L^{c}_{A_0}$, which corresponds to the fixation probability $P_{\text{fix}}(\mathcal{C})$ of the lineage,

$$\begin{aligned}
P_{\text{fix}}(\mathcal{C}) =& \lim_{t\to\infty} L^{c}_{A_0}(t) \\
=& L^{c}_{A_0}(0) + \frac{N_a\,S_a}{1 + 2(\theta_a + \tilde{\theta}_v)} L^{c}_{A_1}(0) + \frac{N_a\,S_a}{1 + 2\theta_a}\hat{L}^{c}_{A_1}(0) \\
&+ \frac{(N_a\,S_a)^2}{(1 + 2(\theta_a + \tilde{\theta}_v))} \times \frac{1}{3 + 4(\theta_a + \tilde{\theta}_v)}\left[L^{c}_{A_2}(0) - L^{c}_{A_{(0;2)}}(0) + 4\theta_a\ell\left(Q_2(0) - Q_{(0;2)}(0)\right)\right] \\
&+ \frac{(N_a\,S_a)^2}{(1 + 2\theta_a)} \times \frac{1}{3 + 4\theta_a}\left[\hat{L}^{c}_{A_2}(0) - \hat{L}^{c}_{A_{(0;2)}}(0) + 4\theta_a\hat{\ell}\left(\hat{Q}_2(0) - \hat{Q}_{(0;2)}(0)\right)\right] \\
&- \frac{(N_v\,S_v)(N_a S_a)}{1 + 2(\theta_a + \tilde{\theta}_v)} \times \frac{1}{1 + 4(\theta_a + \tilde{\theta}_v)}\left[L^{c}_{A_1,V_1}(0) - L^{c}_{A_0,V_2}(0) + 4\theta_v\ell\left(\sqrt{Q_{(0;2)}(0)Q_2(0)} - Q_{(0;2)}(0)\right)\right]
\end{aligned} \tag{S140}$$

The fixation probability of a lineage can be characterized by the state of the antibody and the viral population upon its introduction. The first term in eq. (S140) is the frequency of the antibody lineage at the time of introduction, and is equal to the neutral fixation probability. The terms proportional to the antibody selection coefficient (N_aS_a) measure the relative fitness of the lineage $\mathcal{C}$ to the mean fitness of the population. The terms proportional to the

$(N_a S_a)^2$ measure the relative fitness flux of the lineage $\mathcal{C}$ to the fitness flux of the whole population. The terms proportional to $(N_a S_a) \times (N_v S_v)$ measure the transfer flux from the viral population to the antibody lineage $\mathcal{C}$ relative to the total transfer flux from viruses to the antibody population. $L^{\mathcal{C}}_{A_{(0;2)}}$ and $L_{A_0,V_2} = \langle \rho^{\mathcal{C}} M_{V,2} \rangle$ are respectively the total diversity of binding in the antibody and in the viral population, scaled by the frequency of the lineage $\mathcal{C}$, and determine the fitness flux and transfer flux associated with the whole antibody population. The diversity of binding affinity in viruses is a population observable which affects the lineage fixation probability, as shown in Fig 5.

As mentioned in the main text, the higher viral diversity favors the fixation of broadly neutralizing antibodies for two reasons. First, the larger viral diversity compromises the mean fitness of the resident non-broad antibody population, and makes it easier for the potential BnAb lineage to take over the existing antibody lineages. This effect is captured by terms proportional to $N_a S_a$ in eq. (S140). Second, the transfer flux from the viral population to the lineage with access to the conserved interaction regions (i.e, a lineage with $\hat{E}_0^2/E_0^2 \gg 1$) is small. Therefore, the viral escape from binding to a potential BnAb lineage is less efficient than from the resident non-broad antibody population, which increases the chance of fixation for a potential BnAb lineage. This effect is captured by terms proportional to $(N_a S_a) \times (N_v S_v)$ in eq. (S140).

The approximation used to estimate the fixation probability in eq. (S140) is valid when the effective selection pressure on the lineage (rescaled by the nucleotide diversity) is comparable to the effective pressure on viruses, i.e., $(s_a^{\mathcal{C}} - \sum_{\mathcal{C}'} s_a^{\mathcal{C}'} \rho_{\mathcal{C}'} + \hat{s}_a^{\mathcal{C}} - \sum_{\mathcal{C}'} \hat{s}_a^{\mathcal{C}'} \rho_{\mathcal{C}'})\theta_a \sim s_v \theta_v$, where $s_a^{\mathcal{C}} = N_a S_a (\ell \mathcal{K}_2^{\mathcal{C}})^{1/2}$ and $\hat{s}_a^{\mathcal{C}} = N_a S_a (\hat{\ell} \hat{\mathcal{K}}_2^{\mathcal{C}})^{1/2}$ are the rescaled selection coefficients of the focal lineage $\mathcal{C}$ in the variable and the conserved regions. Fig 5 in the main text shows deviations between analytical expectations from eq. (S140) and the outcome of the Wright-Fisher simulations beyond this approximation regime. Specifically, the analytical predictions become less reliable for the case of an emerging BnAb lineage on the background of a neutralizing resident population, which causes a strong selection imbalance between the two populations. Including the higher order terms of the lineage-specific moments would improve the analytical predictions. However, in the regime of very strong selection, the higher order terms of the series expansion in eq. (S140) become very large (and of alternating sign), so that the fixation probability remains bounded ($0 \leq P_{\text{fix}} \leq 1$). In this regime, we show that substituting the second order lineage-specific moments in eqs. (S117, S124) by their ensemble-averaged expectation in neutrality,

$$L^{\mathcal{C}}_{A_2} - L^{\mathcal{C}}_{A_{(0;2)}} \simeq 4\theta_a \ell \left(Q^{\mathcal{C}}_2(0) - Q^{\mathcal{C}}_{(0;2)}(0) \right), \qquad \hat{L}^{\mathcal{C}}_{A_2} - \hat{L}^{\mathcal{C}}_{A_{(0;2)}} \simeq 4\theta_a \hat{\ell} \left(\hat{Q}^{\mathcal{C}}_2(0) - \hat{Q}^{\mathcal{C}}_{(0;2)}(0) \right) \tag{S141}$$

could provide a more reliable approximation to the fixation probability as opposed to a higher order yet incomplete expansion; see Fig 5 (dashed lines) in the main text.

F Analysis of time-shifted neutralization data

The empirical study by Richman *et al.* [24] provides time-shifted measurements of viral neutralization by a patient's circulating antibodies, as the percent inhibition of viral replication at various levels of antibody dilution compared to an antibody-negative control. The inhibition of the virus for a given concentration of antibodies in the serum $[AB]$ is,

$$I = \frac{[AB]}{[AB] + K} \tag{S142}$$

where K is a constant that equals the antibody concentration which inhibits 50% of viruses. The inhibition can be written in terms of the plasma dilution d_{AB},

$$I(\mathbf{V}(t_1), \mathbf{A}(t_2)) = \frac{d_{AB}(t_2)}{d_{AB}(t_2) + 1/\text{titer}(V_{t_1}, A_{t_2})} \tag{S143}$$

where titer is the reciprocal of antibody dilution where inhibition reaches 50% (IC_{50}). Inhibition by antibodies reduces the replication rate of viruses from the maximum value in the absence of antibodies $r_{\max}$ by a factor $1 - I$, and results in population growth, $N_v(t+1) = r_{\max}(1 - I) N_v(t)$, with a malthusian mean fitness for the viral population $F_v = \frac{1}{t - t_0} \log\left(N_v(t)/N_v(t_0)\right) = \log\left(r_{\max}(1 - I)\right)$. In the patient, the plasma is not diluted i.e., $d_{AB} \sim 1$. Therefore, the viral fitness during infection can be approximately expressed in terms of the neutralization titer $F_v \sim -\log(\text{titer})$. A similar relation between neutralization titers and viral fitness has been previously suggested by Blanquart & Gandon [25].

Additional control experiments show inhibition of a neutralization-sensitive virus (NL43) [24], which we denote by I^*. In the stationary state, we expect that the titers (and fitness) associated with the neutralization-sensitive virus to be comparable across serums of various time-points. However, due to a low antibody response at the initial stages of the infection, the neutralization titers for both autologous viruses and the control NL43 virus grow as the infection progresses; see S7 Fig. In order to account for this non-stationary antibody response, we evaluate the fitness as the relative titers of the autologous viruses and the neutralization-sensitive virus (NL43) at each time-point. We define the relative time-shifted mean fitness of the viral population at time t against the antibody serum sampled at time $t + \tau$ as,

$$F_{V;\tau}(t) = c_0 - \log\left(\text{titer}(V_t, A_{t+\tau}) \Big/ \text{titer}^*(A_{t+\tau})\right) \tag{S144}$$

where $\text{titer}^*(A_{t+\tau})$ is the neutralization titer for NL43 virus against the serum sampled at time $t + \tau$, and c_0 is a constant that relates the relative neutralization titers to the viral fitness.

Fig 4C in the main text shows the time-shifted relative mean fitness $F_{V;\tau}(t)$ averaged over all time-points t, evaluated for two patients (TN-1 & TN-3) from the data provided by Richman *et al.* [24]. Before averaging, we linearly interpolate the raw data to produce equal time shifts (3 months for TN-1 and 6 months for TN-3). Due to the functional form of time-shifted fitness in eqs. (S102-S103), which involves sums of two exponentials, brute force parameter scanning is necessary for a convergent solution. Our results indicate comparable values of nucleotide diversity in antibodies and viruses $\tilde{\theta}_a$ and θ_v. Therefore, we report fits to the simpler analytical forms of time-shifted fitness with common θ's given by eqs. (S102-S103), that use a single exponential function to both sides of the data. Fits are found by scanning parameters and calculating the mean squared errors with appropriate weights due to averaging over equal time-shifts. Each fit contains 4 composite variables which are functions of the underlying evolutionary parameters: (i) nucleotide diversity θ, (ii) selection component of the fitness flux in the viral population $S_v^2 M_{V,2}$, (iii) selection component of the transfer flux from antibodies to viruses, $-S_a S_v M_{A,2}(N_v/N_a)$, and (iv) the constant c_0 in eq. (S144). Assuming that the derivative of the time-shifted fitness function is continuous at the separation time $\tau = 0$, the mean fitness of viruses interacting with their co-residing antibody population can be evaluated dependent on the other fitted parameters, $F_{V;0} = (S_v^2 M_{V,2} - S_a S_v M_{a,2})/4\theta$. The fitted variables are listed below for both patients,

	diversity / month, $\theta \cdot (\text{month}/N_v)$	sel. part of ϕ_V / month, $S_v^2 M_{V,2}$	sel. part of $\mathcal{T}_{A\to V}$ / month, $-S_a S_v M_{A,2}$	offset, c_0
patient TN-1	0.07	0.69	−0.24	−0.24
patient TN-3	0.05	0.20	0	0.52

(S145)

The time-shifted fitness measurements match well with the analytical fits and indicate two distinct regimes of coevolutionary dynamics in the two patients. In patient TN-1, viruses and antibodies experience a comparable adaptive pressure, as indicated by the “S-curve” in Fig 4C (blue line), with $s_v m_{V,2}/(s_a m_{A,2}) = 2.9$. In patient TN-3, adaptation in viruses is much stronger than in antibodies, resulting in an imbalanced shape of the time-shifted fitness curve in Fig 4C (red line). The lower overall neutralization titers in patient TN-3 (S7 Fig) is indicative of such imbalance between the immune response and HIV escape in the patient. It is likely that a longer monitoring of patient TN-3 would capture a stronger antibody response in later stages of infection.

Note that in these studies time is measured in units of months rather than coalescence time of the populations. Estimating the coalescence time-scale in units of months would require analysis of genealogical relations between sequences of antibodies and viruses extracted from each patient over the course of infection, which is not available for this study.

References

[1] Gardiner C (2004) Handbook of Stochastic methods: for physics, chemistry and the natural sciences. Springer, 3rd edition.

[2] Kimura M (1964) Diffusion models in population genetics. J Appl Probab 1: 177–232.

[3] Antonelli PL, Strobeck C (1977) The geometry of random drift I. stochastic distance and diffusion. Adv Appl Probab 9: 238–249.

[4] Fisher RA (1930) The distribution of gene ratios for rare mutations. Proc R Soc Edinb 50: 205–220.

[5] Kimura M (1983) The neutral allele theory of molecular evolution. Cambridge, UK: Cambridge University Press.

[6] Wang S, et al (2015) Manipulating the selection forces during affinity maturation to generate cross-reactive HIV antibodies. Cell 160: 785–797.

[7] Detours V, Perelson AS (1999) Explaining high alloreactivity as a quantitative consequence of affinity-driven thymocyte selection. Proc Natl Acad Sci USA 96: 5153–5158.

[8] Detours V, Perelson AS (2000) The paradox of alloreactivity and self MHC restriction: quantitative analysis and statistics. Proc Natl Acad Sci USA 97: 8479–8483.

[9] Nourmohammad A, Schiffels S, Lässig M (2013) Evolution of molecular phenotypes under stabilizing selection. J Stat Mech Theor Exp 2013: P01012.

[10] Nourmohammad A, Held T, Lässig M (2013) Universality and predictability in molecular quantitative genetics. Curr Opin Genet Dev 23: 684–693.

[11] Higgs PG, Woodcock G (1995) The accumulation of mutations in asexual populations and the structure of genealogical trees in the presence of selection. J Math Biol 33: 677–702.

[12] Good BH, Desai MM (2013) Fluctuations in fitness distributions and the effects of weak linked selection on sequence evolution. Theor Popul Biol 85: 86–102.

[13] Mustonen V, Lässig M (2009) From fitness landscapes to seascapes: non-equilibrium dynamics of selection and adaptation. Trends Genet 25: 111–119.

[14] Mustonen V, Lässig M (2010) Fitness flux and ubiquity of adaptive evolution. Proc Natl Acad Sci USA 107: 4248–4253.

[15] Fisher RA (1930) The genetical theory of natural selection. Oxford University Press, USA, 1st edition.

[16] Kosmrlj A, Jha AK, Huseby ES, Kardar M, Chakraborty AK (2008) How the thymus designs antigen-specific and self-tolerant T-cell receptor sequences. Proc Natl Acad Sci USA 105: 16671–16676.

[17] Kosmrlj A, Chakraborty AK, Kardar M, Shakhnovich EI (2009) Thymic selection of T-cell receptors as an extreme value problem. Phys Rev Lett 103: 068103.

[18] de Haan L, Ferreira A (2006) Extreme value theory: an introduction. New York: Springer US.

[19] Mustonen V, Lässig M (2007) Adaptations to fluctuating selection in Drosophila. Proc Natl Acad Sci USA 104: 2277–2282.

[20] Held T, Nourmohammad A, Lässig M (2014) Adaptive evolution of molecular phenotypes. J Stat Mech Theor Exp 2014: P09029.

[21] Nourmohammad A, Rambeau J, Held T, Berg J, Lässig M (2015) Pervasive adaptation of gene expression in Drosophila. arXiv : q-bio/1502.06406v2.

[22] Price GR (1970) Selection and covariance. Nature 227: 520–521.

[23] Desai MM, Fisher DS (2007) Beneficial mutation–selection balance and the effect of linkage on positive selection. Genetics 17: 385–394.

[24] Richman DD, Wrin T, Little SJ, Petropoulos CJ (2003) Rapid evolution of the neutralizing antibody response to HIV type 1 infection. Proc Natl Acad Sci USA 100: 4144–4149.

[25] Blanquart F, Gandon S (2013) Time-shift experiments and patterns of adaptation across time and space. Ecol Lett 16: 31–38.
